# Multi-omics analyses reveal dynamic interactions between DNA methylation and transcriptional regulation during black raspberry ripening

**DOI:** 10.1038/s42003-026-11289-3

**Published:** 2026-11-12

**Authors:** Wei-Hsun Hsieh, Yu-Hung Hung, Jian-Hui Zhang, Qirui Cui, Han-Yi Chen, Liang-Peng Lin, Meng-Hsun He, Yen-Ching Wang, Yu-Yu Ho, James Ballington, Gina Fernandez, Penelope Perkins-Veazie, Brandon H. Le, Xu Li, Tzung-Fu Hsieh, Jer-Young Lin

**Affiliations:** 1 Agricultural Biotechnology Research Center, Academia Sinica, Taipei, Taiwan, ROC; 2 Biotechnology Research Center in Southern Taiwan, Academia Sinica, Tainan City, Taiwan, ROC; 3 Department of Plant and Microbial Biology, North Carolina State University, Raleigh, NC USA; 4 Plants for Human Health Institute, North Carolina State University, North Carolina Research Campus, Kannapolis, NC USA; 5 Department of Horticultural Science, North Carolina State University, Raleigh, NC USA; 6 Institute for Integrative Genome Biology Bioinformatics Core Facility, University of California, Riverside, CA USA; 7 Institute of Tropical Plant Sciences and Microbiology, National Cheng Kung University, Tainan, Taiwan, ROC; 8 Biotechnology Center, National Chung Hsing University, Taichung, Taiwan, ROC

**Keywords:** DNA methylation, Transcriptomics, Plant development

## Abstract

Fruit ripening is a complex developmental process involving coordinated changes in gene expression, metabolism, and epigenetic regulation. How DNA methylation dynamics influence gene transcription and metabolite accumulation during black raspberry (*Rubus occidentalis*) fruit ripening remains poorly understood. Here, we performed developmental-series transcriptome, methylome, and metabolome analysis; the results from this analysis reveal extensive epigenetic reprogramming during black raspberry fruit ripening. Fruit tissues exhibit globally higher DNA methylation than leaves, particularly in CHG and CHH contexts. Local methylation in all cytosine contexts progressively decreases in promoter regions during ripening, whereas CHG and CHH methylation increase in transposon-rich regions. Two primary methylation transitions—promoter hypomethylation and CHH hypermethylation—govern transcriptional shifts in genes involved in ripening processes. Methyl-binding transcription factors with activation potential likely promote CHH hypermethylation-linked transcriptional activation. Multi-omics integration reveals that coordinated anthocyanin accumulation parallels expression of biosynthetic and regulatory genes within coherent networks. Elevated non-CG methylation in heterochromatin coincides with increased transcription of histone variants mediating chromatin compaction, suggesting chromatin remodelers fine-tune accessibility for methyltransferases. Our study highlights both genome-wide and locus-specific epigenetic reprogramming and demonstrates a coordinated interplay between DNA methylation and transcriptional regulation during black raspberry fruit ripening.

## Introduction

Fleshy fruit ripening is characterized by physiological modifications in color, texture, sugar content, aroma, and flavor, enhancing their attractiveness to seed-dispersal vectors. This transition is tightly controlled, and misregulation results in deleterious consequences^1^. Fruits have traditionally been categorized into climacteric and non-climacteric groups based on fruit physiology during ripening. Climacteric fruits, such as tomato, exhibit a distinct increase in respiration and a concomitant peak in ethylene production that triggers the ripening process. The hormone responses, genetic factors, and molecular mechanisms underlying ripening processes in climacteric fruits are relatively well documented^1^. By contrast, non-climacteric fruit ripening, such as strawberry, grape, and citrus, remains poorly understood. Recent studies have begun to uncover molecular mechanisms underlying this process. Non-climacteric fruits typically lack these characteristic bursts, showing no significant surge in ethylene or respiration as they mature. Emerging research suggests that ripening in non-climacteric fruits is associated with multiple hormones, such as abscisic acid (ABA), gibberellin (GA), and auxin^1^. The fruit ripening process is also coordinated by transcription factors (TFs) and epigenetic modifications^2,3^. In tomato, the TF *CNR* gene is silenced in the colorless non-ripening (*Cnr*) mutant due to promoter hypermethylation, arresting fruit development^4^. Ripening Inhibitor (RIN), a MADS-box TF, activates numerous ripening-related genes marked by promoter hypomethylation^5^. Disruption of the DNA demethylase *SlDML2* gene in tomato delays ripening^6,7^, suggesting that active DNA demethylation underlies this developmental program. Alterations in DNA methylation at MYB loci have been associated with changes in anthocyanin biosynthesis in apple and pear^8,9^. These studies demonstrated that changes in DNA methylation play a pivotal role in fruit ripening regulation.

DNA methylation regulates important cellular processes, such as differentiation, gene transcription, transposon silencing, and genomic imprinting^10^. In euchromatin, DNA methylation at promoters repress transcription by inhibiting TF binding or by recruiting repressors to target loci. In TE-rich heterochromatin, methylation facilitates chromatin compaction and histone modifications, which recruit chromodomain methyltransferases for non-CG methylation, forming a positive feedback loop to reinforce silencing^11^. DNA methylation can also positively regulate transcription by recruiting reader proteins that enhance chromatin accessibility transcriptional machinery. For example, the *Repressor of Silencing 1* (*ROS1*) gene depends on the RdDM methylation to activate its transcription^12,13^,which involves methylation readers SUVH1 and SUVH3 that further recruit transcriptional activators^14,15^.

Shifts in DNA methylation during ripening occur in climacteric and non-climacteric fruits. Global hypermethylation was observed in pear, apple, orange, and grape, while hypomethylation also occurs in tomato and strawberry^16–19^. Hypomethylation in strawberry is linked to suppression of the RdDM pathway, whereas enhanced DNA demethylase gene expression promotes ripening in tomato^5,20^. Hypermethylation in apple and orange results from downregulation of DNA demethylase genes^17,19^. Thus, fruit-specific DNA methylation changes are driven by species-specific mechanisms.

**Fig. 1:**
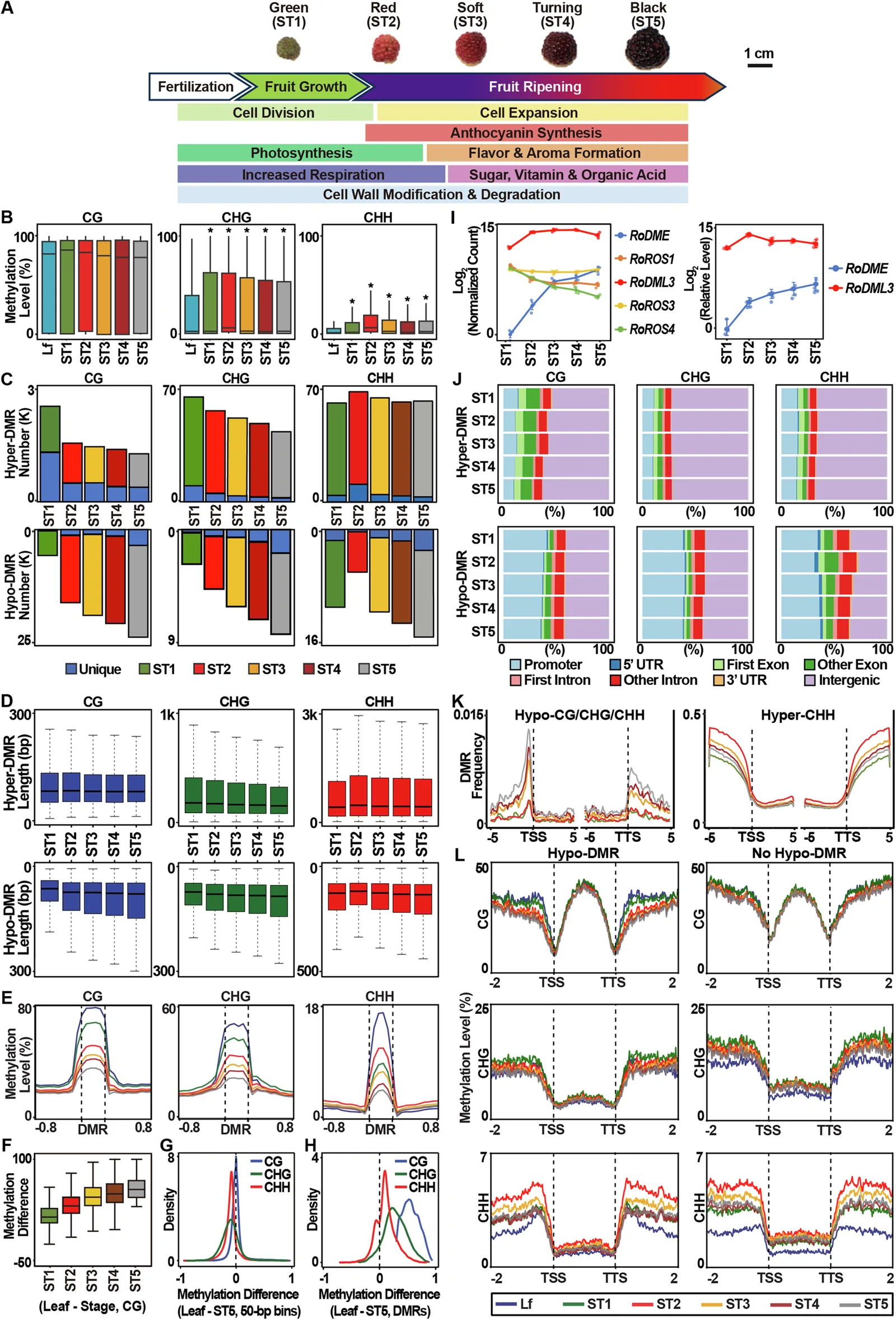
Schematic representation of black raspberry fruit developmental stages and associated DNA methylation features during ripening. **A** Black raspberry fruit ripening stages and major developmental events. Scale bar: 1 cm. **B** Box plots of DNA methylation levels of 50-bp windows across the genome in the indicated tissue and fruit stage. The *n* values indicate the numbers of 50-bp windows included in each box plot after removal of windows with missing values. * Indicates significant difference relative to the leaf stage (by two-sided Wilcoxon test with FDR < 0.05 and fold change >1.2X). **C** Number of hyper- and hypo-DMRs at each fruit ripening stage compared with leaf. **D** Lengths of differentially methylated regions (DMRs) in hypo- and hyper-DMRs during black raspberry fruit ripening. Leaf tissue was used as the reference tissue. The *n* values represent the numbers of DMRs included in each box plot and correspond to the DMR number shown in (**C**). **E** DNA methylation patterns of gene-proximal CG, CHG, and CHH hypo-DMRs, respectively, derived from ST5-to-leaf comparison, across black raspberry fruit ripening and leaves. **F** DNA methylation levels of CG hypo-DMRs identified in the black stage compared to the leaf are shown across all ripening stages. The *n* value represents the number of CG hypo-DMRs included in each box plot, corresponding to the CG hypo-DMR number shown in (**C**). **G** Genome-wide DNA methylation differences between the black stage (ST5) and leaf, calculated using 50-bp tiling windows. **H** DNA methylation differences between ST5 and leaf for each methylation context, for ST5-vs-leaf CG hypo-DMRs. **I** Temporal expression patterns of DNA demethylase genes across ripening stages. Left panel: Log_2_-transformed average normalized read counts, based on *n* = 3 biologically independent samples for ST1–ST3 and ST5, and *n* = 2 for ST4. Right panel: relative expression levels of *RoDML3* vs *RoDME* assessed by qRT-PCR, based on *n* = 4 biologically independent samples for both genes at ST1–ST5, except for *RoDME* at ST1 (*n* = 3). Values represent means; individual data points represent biological replicates, and error bars indicate standard deviations of the biological replicates (one-way ANOVA, *p* = 0.0063 for ST1, *p* < 0.001 for ST2-ST5). **J** Distribution of hyper and hypo DMRs in different genomic features during black raspberry fruit ripening. **K** DMR frequency in genic regions. Hypo-CG/CHG/CHH DMRs used here represent the intersection of all three sequence contexts. **L** DNA methylation profiles across genic regions in genes associated (2489 genes) or not associated with (randomly selected 2479 genes) hypo-CG/CHG/CHH DMRs (intersection of all three sequence contexts).

Black raspberry (*Rubus occidentalis*) fruits have high levels of anthocyanins, contributing to its exceptional antioxidant capacity and associated health benefits^21^. The berry is an aggregate of individual drupelets, each anatomically analogous to a cherry, featuring a hard endocarpic seed (pyrene) surrounded by fleshy mesocarp and a thin outer exocarp skin^22–24^. Fruit development progresses from early stage of intense cell division to reduced proliferation as the embryo matures, culminating rapid fruit expansion driven by cell enlargement (Fig. 1A). To date, *Rubus* ripening research primarily focused on transcriptomic and metabolic profiling, identifying physiological changes and TFs involved in anthocyanin biosynthesis^25–27^. Despite its nutritional importance, how DNA methylation regulates gene expression in fruits remains largely unexplored.

The black raspberry genome size (V3) is approximately 290 Mb, with long terminal repeat (LTR) retrotransposons accounting for 32.6% of the genome^28^. We profiled developmental series of transcriptome, methylome, and targeted metabolome to characterize molecular events underlying fruit ripening. Global DNA methylation in fruit is reprogrammed, with overall levels significantly higher than in leaves, particularly in CHG and CHH contexts. Transcriptional reprogramming is associated with two predominant types of local methylation changes: promoter-associated CG/CHG/CHH hypomethylation and CHH hypermethylation, both correlate with altered gene expression relevant to fruit physiological process. Multi-omics integration revealed correlated accumulation of anthocyanins with co-expression of biosynthetic and regulatory genes interconnected within defined gene regulatory networks.

Elevated non-CG methylation in fruit heterochromatin coincides with increased transcription of histone variants required for compacting heterochromatin that is inaccessible to DNA methyltransferases and may reflect a finely tuned regulatory epigenetic state that permits accessibility to methyltransferases despite the presence of compaction-associated histones. Our study reveals the genetic and epigenetic programs that orchestrate fruit maturation and anthocyanin biosynthesis in black raspberry.

## Results

### Black raspberry fruit methylomes throughout development and ripening

We generated single-base resolution DNA methylation profiles of black raspberry fruit across five developmental stages using bisulfite sequencing (BS-Seq), capturing the full progression of the methylation landscape of the leaves and reproductive tissues. These stages were designated as green (ST1), red (ST2), soft (ST3), turning (ST4), and black (fully ripe; ST5) (Fig. 1A). We generated ~1.06 billion BS-Seq reads from all fruit stages and leaf, obtaining 8.1–21.9× coverage of each strand of the genome (Supplementary Data 1A, average coverage of 24.6× and a median of 23.3×). We identified 97.1–99.5 million cytosines, representing 89.0–91.2% of all cytosines in the genome. Across the fruit samples, the average methylation levels were ~55% (CG), ~24% (CHG), and ~5% (CHH, H=C, A or T), corresponding to ~64%, ~39%, and ~20% of cytosines methylated in each context (Supplementary Data 1B). BS-Seq data from replicates were in excellent agreement with each other (correlation coefficients >0.94, Fig. S1). Principal component analysis (PCA) also grouped replicates together in each context (Fig. S1B). Taken together, these results indicate that our datasets represent unbiased and reproducible profiles of a temporal DNA methylation changes throughout fruit ripening.

### Global DNA methylation levels in fruit ripening are higher than in leaf

For global DNA methylation analysis, the number and fraction of methylated cytosines in 50-bp tiling windows were assessed. Relative to leaf, the fruit undergoes genome-wide DNA methylation changes, showing significant increases in CHG and CHH methylation (two-sided Wilcoxon test with FDR correction, Fig. 1B, Fig. S2A, and Supplementary Data 2). Whereas the fold change in CG methylation between these tissues is relatively small (1–2%), the differences in CHG and CHH contexts are more substantial (over 20%), highlighting the context-specific methylation reprogramming (Supplementary Data 2). Compared with the green (ST1), CG methylation is slightly reduced in later stages, while CHG methylation declines and CHH methylation increases (Fig. 1B and Fig. S2B), echoing the whole genome average methylation levels (Fig. S2C). Symmetric methylation sites (CG and CHG) gradually decline from ST1 to ST5, whereas the number of methylated CHH sites peaks at ST2 and decreases during later stages; the average methylation levels remain steady (Fig. S2C, D). Together, these results demonstrate that genome-wide methylation patterns undergo reprogrammed during fruit development and are elevated compared to leaves, highlighting distinct methylation dynamics in the fruit.

### Local DNA methylation patterns are changed across fruit ripening

Next, we investigated changes in local DNA methylation patterns (differentially methylated regions, DMRs; CGmapTools^29^: *p* < 0.05, methylation differences of CG > 20%, CHG > 10%, and CHH > 8%). To comprehensively inspect the occurrence of DMRs in fruit, DNA methylation levels in leaf were used as baseline references. We found that there were more hypo-DMRs than hyper-DMRs in the CG context, whereas in the non-CG contexts, hyper-DMRs outnumbered hypo-DMRs (Fig. 1C and Fig. S3). The number of hyper-DMRs decreased in the CG and CHG contexts, while the number of hypo-DMRs increased during fruit ripening (Fig. 1C and Fig. S3). Remarkably, most DMRs occurred in the same loci, with very few being unique in pairwise comparison. The lengths of hypo-DMRs in all three cytosine contexts increased as the fruit developed, while those in hyper-DMRs remained steady or slightly decreased (Fig. 1D). The increase in length and limited unique new hypo-DMRs in successive stages indicated that most demethylations occurred on the same loci.

### Progressive active DNA demethylation at specific loci during fruit ripening

We examined DNA methylation levels within the ST5-vs-leaf hypo-DMRs across all stages and found a continued decline in methylation in each cytosine context as ripening progressed, indicating that these hypo-DMRs progressively lose DNA methylation during ripening (Fig. 1E). Consequently, the CG methylation difference between fruit and leaves continued to increase in CG hypo-DMRs as ripening progressed (Fig. 1F). We next examined the distribution of methylation difference of 50-bp tiling windows. Genome-wide, CHG and CHH methylation levels of the 50-bp bins were generally higher in ST5 relative to leaves (density peaks on negative side), while CG methylation levels were similar in both tissues (Fig. 1G). However, the CG, CHG, and CHH methylation levels within the ST5 CG hypo-DMRs were substantially lower relative to leaves (Fig. 1H), indicating that these ST5 CG hypo-DMRs were demethylated in all cytosine contexts. These local extensive DNA demethylation most likely results from active DNA demethylation, consistent with reports from earlier studies^5–7,30^. Supporting this, we found various demethylase genes are expressed during ripening. Among them, *RoDML3* exhibited the highest expression level, suggesting that it is likely the major player responsible for local demethylation during ripening (Fig. 1I and Supplementary Data 3A). Notably, BLASTP analysis identified *RoDML3* as the direct homolog of the tomato *SlDML2*, a validated functional demethylase in tomato ripening^6^ (Supplementary Data 4). Our results indicated that black raspberry fruit involves locus-specific reductions in CG, CHG, and CHH methylation mediated by active DNA demethylation.

### Hypo-DMRs are located primarily in promoter and gene body regions

The hyper- and hypo-DMRs are preferentially distributed across different genomic regions. Most hypo-DMRs in all three cytosine contexts were in genic regions (Fig. 1J), among which more than half were in promoters; by contrast, most hyper-DMRs were found in intergenic regions (Fig. 1J). Non-CG hyper-DMRs were co-localized in chromosomal regions enriched for LTR retrotransposons (Fig. S4). Consistent with the decrease in non-CG methylation levels in the CG hypo-DMRs, many non-CG hypo-DMRs overlapped with CG hypo-DMRs (Fig. S5). The frequency of CG/CHG/CHH hypo-DMRs was greater in regions upstream and proximal to the transcription start site (TSS) than in regions downstream of the transcription termination site (TTS), and DMRs in both ends diminished progressively in the distal regions (Fig. 1K). By contrast, CHH hyper-DMRs accumulated distal to the TSS and TTS and increased successively into the distal regions (Fig. 1K). These distinct spatial patterns across genic regions indicate that various epigenetic modifications may shape regulatory landscapes flanking genic regions in a context-dependent manner. Interestingly, genes containing CG/CHG/CHH hypo-DMRs exhibited lower DNA methylation levels around both the TSS and TTS compared with genes lacking these DMRs (Fig. 1L), suggesting distinct molecular mechanisms were involved in establishing their DNA methylation states.

Overall, DNA methylation landscapes differed substantially between leaf and fruit. Early in fruit development, DNA methylation declined in specific promoters and genic regions, presumably mediated by the DNA demethylases active in early ripening. As ripening progressed, active demethylation continued and expanded in the same loci, as well as at newly targeted sites (Fig. S6). By contrast, relative to leaf, non-CG methylation increased mainly in non-genic regions, particularly within repetitive sequences (Fig. S4). Our results suggest that black raspberry fruit ripening involves multiple mechanisms of DNA methylation reprogramming.

### Extensive transcriptomic reprogramming occurs at the soft fruit stage

**Fig. 2:**
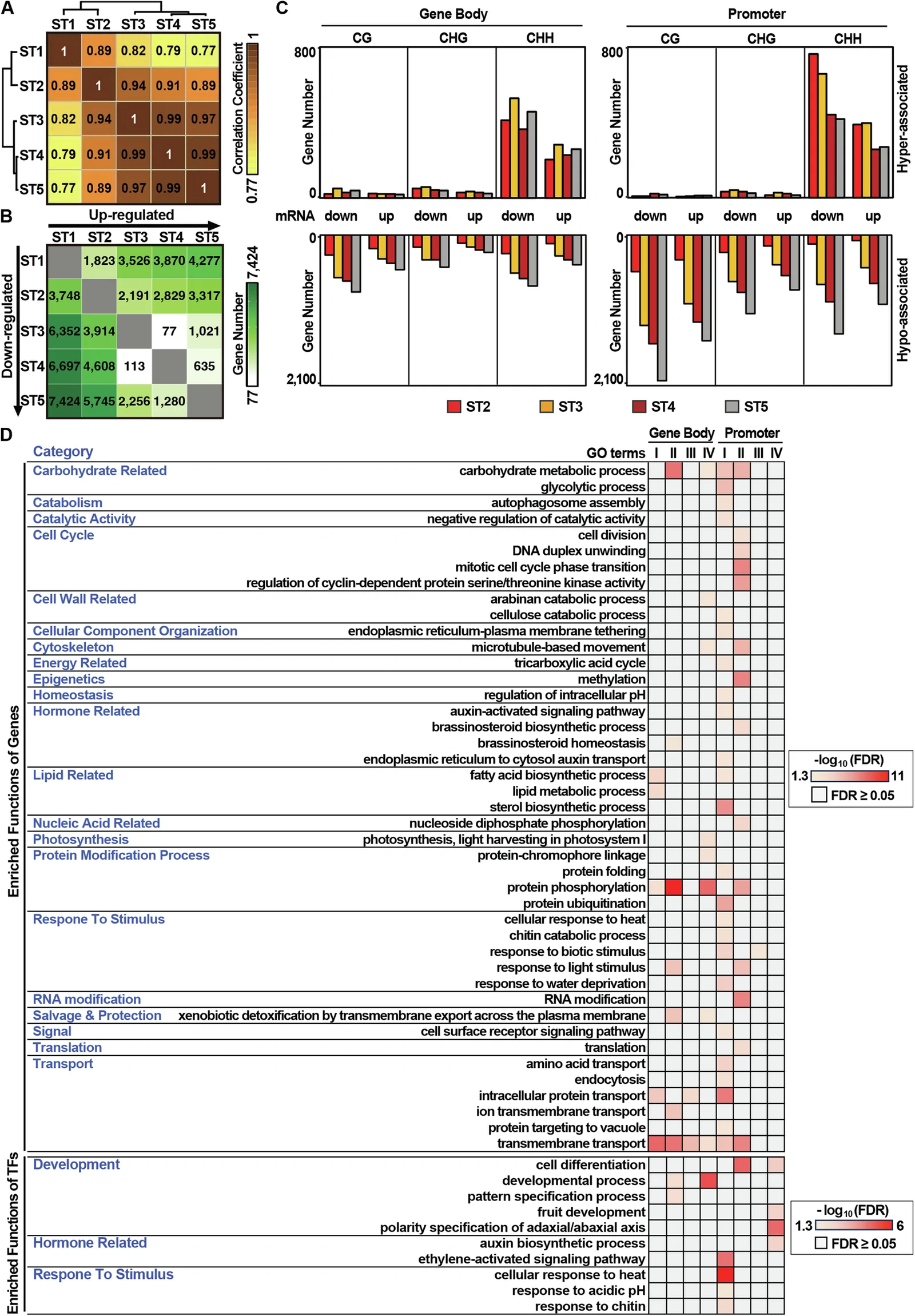
Gene expression dynamics and the overlap of differentially expressed genes (DEGs) and differentially methylated genes (DMGs), along with their enriched functional categories during black raspberry fruit ripening. **A** Hierarchical clustering of Pearson correlation coefficients of mRNA populations illustrating the association of ripening stages. **B** Differentially expressed genes in a pairwise comparison. The numbers in the matrix represent the DEG numbers. The left side of the diagonal represents genes down-regulated in one stage on the Y-axis compared to the X-axis. The right side of the diagonal represents genes up-regulated in one stage on the X-axis compared to the Y-axis. **C** Bar plot summarizing genes that are both DEGs and DMGs, with DMRs located in promoter or gene body regions, using ST1 as the reference. mRNA down: genes downregulated relative to ST1; mRNA up: genes upregulated relative to ST1. **D** Enriched Gene Ontology (GO) biological process terms for DEGs and differentially expressed transcription factors associated with CG, CHG, and CHH hypo-DMRs, as well as CHH hyper-DMR in promoter or gene body regions. I: CG/CHG/CHH hypo-DMR associated with up-regulated genes; II: CG/CHG/CHH hypo-DMR associated with down-regulated genes; III: CHH hyper-DMR associated with up-regulated genes; IV: CHH hyper-DMR associated with down-regulated genes. CG/CHG/CHH hypo-DMRs used here represent the union of hypo-DMRs from all three sequence contexts.

To understand transcriptomic changes that drive black raspberry fruit ripening, we analyzed genome-wide gene expression patterns across five fruit stages. We inspected the correlations of transcriptomes between bio-replicates using PCA to demonstrate that bio-replicates are closely correlated with each other and that transcripts between different stages are distinct (Fig. S7, one outlier from ST4 was excluded from further analysis). We applied unsupervised hierarchical clustering to inspect the relationships of whole-genome transcripts between fruit ripening stages (Fig. 2A). They can be divided into two groups: an early group from green to red (ST1 and ST2) and a late group from soft to black (ST3 to ST5). Notably, soft (ST3) is distal from the other two stages (ST4 and ST5) in the late group, representing a transition stage marked by a distinct mRNA population. To uncover the gene activities in each stage compared to others, we applied DESeq2 (FDR < 0.05, fold change >2X) to identify differentially expressed genes (DEGs). A total of 14,544 genes (representing 43.7% of the annotated genome) are differentially expressed, with a greater number of genes down-regulated than upregulated (Fig. 2B). In sequential stage-to-stage comparisons, more DEGs were identified during the early transition stages (from ST1 to ST2 and ST2 to ST3) than in the later transition stages (ST3 to ST4 and ST4 to ST5), indicating that a larger-scale transcriptomic change occurs during early fruit development. Notably, the transition from ST2 to ST3 was marked by the most pronounced transcriptional shift, with 2191 genes upregulated and 3914 genes downregulated relative to ST2. To uncover the biological processes regulated by transcriptional programs during fruit ripening, we performed Gene Ontology (GO) analysis on DEGs between consecutive developmental stages (Fig. S8 and Supplementary Data 5). Genes upregulated at the soft stage (ST3) were found to be enriched in biological functions including fatty acid biosynthesis, glycolysis, flavonoid biosynthesis, and ABA signaling, while downregulated genes were associated with processes such as photorespiration and mitochondrial organization. These functions reflect many physiological signatures of the fruit ripening processes. Together, these transcriptome analyses revealed two transcriptionally distinct phases during black raspberry fruit ripening, suggesting extensive transcriptional reprogramming.

### The activities of a subset of genes are associated with DNA methylation changes

To examine the relationship between local DNA methylation changes and gene transcription activities, we identified differentially methylated genes (DMGs) as those harboring DMRs in the promoter (1.5 kb upstream of the TSS) and gene body. By intersecting DMGs with DEGs, we identified a subset of genes whose expression changes were associated with alterations in promoter or gene body methylation (Fig. 2C and Supplementary Data 6). The analysis revealed that DEGs associated with hypo-DMRs in all cytosine contexts or with CHH hyper-DMRs far exceeded those associated with CG or CHG hyper-DMRs, suggesting that CG/CHG/CHH hypomethylation and CHH hypermethylation substantially contribute to the transcriptional dynamics in black raspberry fruits. Notably, we observed that hypo-DMRs in promoter or gene body regions were associated with both up- and down-regulation of gene expression. This dual regulatory pattern resembles the finding in *Arabidopsis* endosperm where local demethylation can lead to either transcriptional activation or repression^31^.

We examined major biological functions of DEGs-associated DMGs in promoter or gene body regions (Fig. 2D and Supplementary Data 7). Among the upregulated genes with CG/CHG/CHH hypo-DMRs, enriched functions included glycolysis, lipid metabolism, and transport, as well as hormone-related processes, such as auxin (e.g., *ARF1*, *LRP1,* and *PILS5*). By contrast, downregulated genes with CG/CHG/CHH hypo-DMRs were primarily associated with cell cycle regulation and hormone-related processes, such as the brassinosteroids (Fig. S9A). Photosynthesis (e.g., *CRD1* and *DXS1*) was enriched in downregulated genes with CHH hyper-DMRs. Additionally, both up and downregulated DEGs linked to hypo-CG/CHG/CHH DMRs or hyper-CHH DMRs were enriched for overlapping functions, including carbohydrate metabolism (e.g., *PGF10*, *6PGD,* and *BGLU17*), protein phosphorylation (e.g., *ABC1-like*, *SERK1,* and *Haspin*), and transport (e.g., *ABCG40* and *DTX44*) (Fig. S9A). Interestingly, downregulated DEGs associated with CHH hyper-DMRs in the gene body displayed a broader spectrum of functional categories than their upregulated counterparts, suggesting that CHH hypermethylation can influence transcription bidirectionally, with repression as the predominant effect. We further analyzed differentially expressed TF genes associated with DNA methylation changes in promoter or gene body regions (Fig. 2D and Supplementary Data 8), revealing that the major functions are related to development, response to stimulus, and hormone-related processes, such as auxin and ethylene.

Putative regulators, such as *NOR*, *CNR*, and *TDR4,* exhibited expression changes linked to local DNA methylation alterations (Fig. S9B). Like the tomato *NOR* gene, *RoNOR* is also highly expressed during fruit development, and the increase in transcription is associated with promoter CG hypomethylation as the fruit ripens (Fig. S9B–D), consistent with tomato *NOR* being a direct target of *SlDML2*^32^. The expression of *RoCNR* and *RoTDR4* are also associated with methylation changes, leading to transcriptional repression during early fruit development (Fig. S9B–D). This differs from the expression patterns of their tomato or bilberry counterparts exhibiting increased transcription during ripening^33–35^, likely reflecting the roles in species-specific physiology regulation during fruit development^3,36^. Together, these results show that DNA methylation changes are associated with transcriptional reprogramming of genes related to black raspberry fruit ripening.

### Transcriptional dynamics of epigenetic regulators promoting methylated gene expression and DNA methylation

**Fig. 3:**
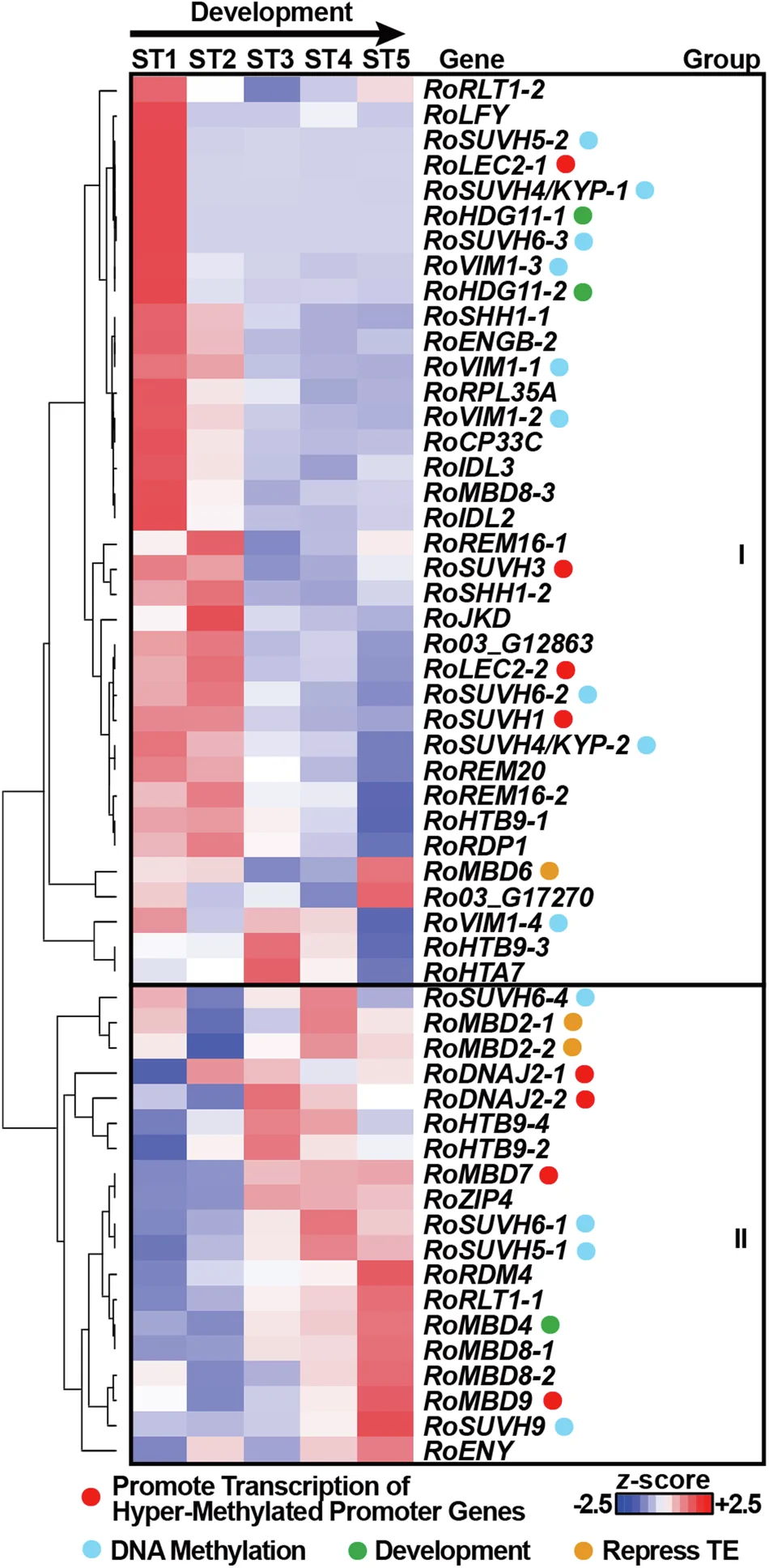
Expression profiles of genes encoding methyl-binding transcription factors and proteins. Unsupervised hierarchical clustering reveals two major transcript groups across black raspberry ripening stages. Genes marked with different colored circles represent distinct biological functions. Gene expression normalized count values are shown as z-scores calculated for each gene across samples.

DNA methylation is traditionally associated with repression, and indeed, we observed hyper-CHH DMRs are predominantly associated with downregulation. Interestingly, we also observed that elevated CHH methylation can coincide with gene activation enriched in specific functions (Fig. 2D). This pattern aligns with recent studies showing that specific methyl-binding TFs and proteins (e.g., LEC2, DNAJ2, SUVH1, SUVH3, and MBD9) can bind methylated DNA and promoting gene expression in target loci^15,37–39^. Conversely, other methyl-binding TFs and proteins, such as members of the REM family, VIM1, and SUVH4/5/6, can recruit DNA methylation machinery (e.g., RdDM complex or DNA methyltransferases) to establish locus-specific methylation for transcriptional silencing^37,40^. These proteins are known to maintain repressive states through self-reinforcing loops formed between histone modifications and DNA methylation^10,11^. Transcriptional profiling of methyl-binding TFs and proteins during fruit ripening revealed two expression patterns (Fig. 3 and Supplementary Data 3B): an early group (e.g., *RoLEC2*, *RoSUVH1/3*, and *REM* TF) and a late group (e.g., *RoMBD9* and *RoDNAJ2*). These results suggested that sequence-specific TFs and methyl-binding proteins may direct transcriptional activation or DNA methylation in a locus- and stage-specific manner during fruit ripening.

### Anthocyanin compounds are predominantly synthesized from the red through the black stages

**Fig. 4:**
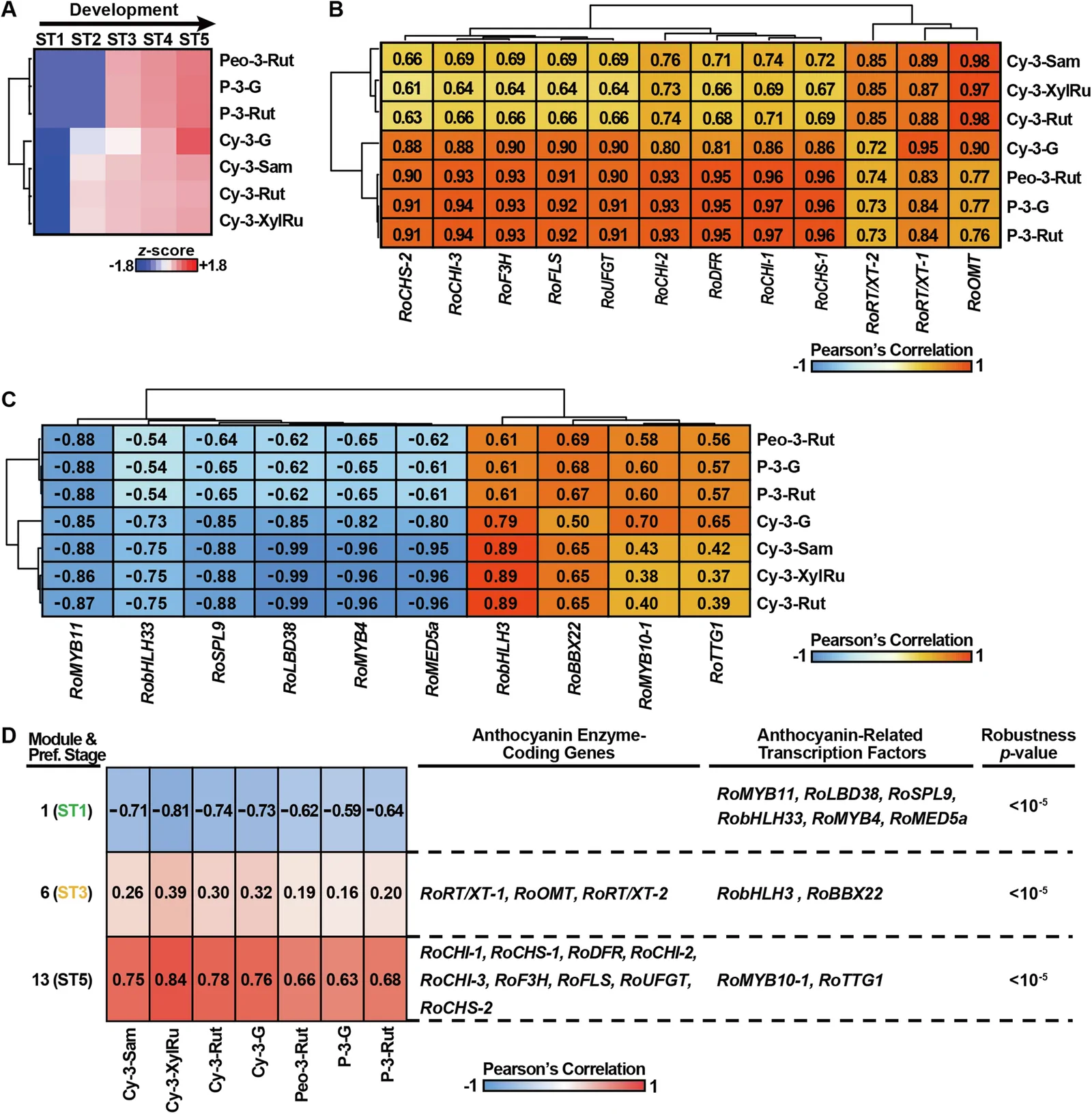
Metabolome analysis of anthocyanin accumulation. **A** Relative levels of anthocyanin compounds clustered by unsupervised hierarchical clustering across ripening stages. Metabolite abundances are shown as z-scores calculated for each metabolite across samples. **B** Pearson correlation coefficients between transcript levels of 12 enzyme-coding genes in the anthocyanin biosynthetic pathway and the relative abundance of seven anthocyanin compounds. **C** Pearson correlation coefficients between transcript levels of transcription factors regulating anthocyanin biosynthetic genes and the abundance of seven anthocyanin compounds. **D** Module–trait heatmap showing the correlation between gene expression modules and anthocyanin content. Color gradients indicate the strength and direction of the correlations. Cy-3-Sam, cyanidin 3-sambubioside; Cy-3-XylRu, cyanidin 3-xylosylrutinoside; Cy-3-Rut, cyanidin 3-rutinoside; Cy-3-G, cyanidin 3-glucoside; Peo-3-Rut, peonidin 3-rutinoside; P-3-G, pelargonidin 3-glucoside; P-3-Rut, pelargonidin 3-rutinoside. Module robustness was evaluated by the permutation test, showing these three modules are significantly robust with *p* < 10⁻⁵.

To monitor the temporal accumulation patterns of anthocyanin species, we profiled metabolomes throughout black raspberry fruit ripening. We detected 89 metabolites, including seven anthocyanin species known to be produced in black raspberry fruit^41^ (Supplementary Data 9). We used unsupervised hierarchical clustering to examine the relative abundance of detected anthocyanins across ripening stages, identifying two main groups: (i) those that increase substantially at red stage (ST2; cyanidin 3-glucoside, cyanidin 3-sambubioside, cyanidin 3-xylosylrutinoside, and cyanidin 3-rutinoside), and (ii) those that rise at soft stage (ST3; pelargonidin 3-glucoside, pelargonidin 3-rutinoside, and peonidin 3-rutinoside) (Fig. 4A). Together, these results suggest that anthocyanin biosynthesis is developmentally regulated and primarily initiated at the red or soft stage.

### Anthocyanin accumulation correlates with the expression of biosynthetic genes and their regulatory transcription factors

To understand how anthocyanin accumulation is transcriptionally modulated during black raspberry ripening, we examined transcript levels of anthocyanin biosynthetic genes in parallel with the relative abundance of seven detected anthocyanins across stages (Fig. 4B and Fig. S10). The putative enzymes involved in the metabolic pathways of these seven anthocyanins were deduced based on known pathway genes in other plant species^42^. Using a correlation coefficient cutoff of *r* ≥ 0.8, we identified 12 transcripts of enzyme-coding genes that are strongly associated with anthocyanin levels: early biosynthetic genes (EBGs), such as chalcone synthase (CHS), chalcone isomerase (CHI), and flavanone 3-hydroxylase (F3H), and late biosynthetic genes (LBGs), such as flavonoid 3-O-glucosyltransferase (UFGT) and O-methyltransferase (OMT) (Fig. 4B and Fig. S10). We further investigated whether the expression patterns of the TFs known to regulate anthocyanin biosynthetic genes are also correlated with anthocyanin accumulation. Using the same approach, we identified several TF transcripts that are either strongly positively or negatively correlated (*r* ≥ 0.8 or *r* ≤ – 0.8) with anthocyanin levels (Fig. 4C, Supplementary Data 10, and Fig. S10). For example, *RobHLH3*, a putative component of the MBW complex that targets LBGs^43^, was strongly positively correlated with anthocyanin content, while *RoSPL9*, a negative regulator of anthocyanin production that destabilizes the MBW complex^44^, exhibited a strong negative correlation with anthocyanin levels (Fig. 4C and Fig. S10). Finally, we examined the DNA methylation patterns of anthocyanin pathway genes and found that several of them, including *RoFLS* and *RoCHS-1*, show methylation changes associated with altered transcript levels. A similar pattern was observed in the TFs regulating these pathways, such as *RoMYB10-1* and *RoMYB11* (Fig. S10). Together, the results suggest that anthocyanin accumulation and the expression of anthocyanin biosynthetic genes are coordinated, and their transcriptional regulation may be involved in modulating anthocyanin levels during black raspberry fruit ripening.

### Interconnecting anthocyanin biosynthetic genes and their transcription factors within gene networks coordinating anthocyanin accumulation

To characterize the gene regulatory events programing the black raspberry fruit ripening and governing anthocyanin accumulation, we first inferred gene regulatory network using weighted gene co-expression network analysis (WGCNA) of highly regulated genes (DESeq2 FDR < 0.05 and >2-fold change). A total of 14,457 genes were organized into 14 gene regulatory modules. To understand the temporal relationships between gene modules and ripening stages, we calculated correlation coefficients and performed hierarchical clustering on the resulting *r* values (Fig. S11). This revealed stage-specific gene modules, such as module 10, which were strongly associated with the black stage. To further uncover the genetic architecture of anthocyanin biosynthesis, we integrated anthocyanin accumulation patterns with inferred gene networks to assess the relationships between gene modules and anthocyanin contents. We found that the seven anthocyanins are highly associated with modules 6 and 13 (Fig. 4D). Specifically, the identified anthocyanin biosynthetic genes (Fig. 4B) were enriched in module 6 (Fig. 4D), associated with ST3 (the soft stage; Fig. S11), and in module 13 linked with ST5 (the black stage; Fig. S11). These two modules contained four TFs (*RobHLH3*, *RoBBX22*, *RoMYB10-1,* and *RoTTG1*) (Fig. 4D) whose expression levels were positively correlated with anthocyanin accumulation (Fig. 4C). Therefore, the consistent positive associations observed between mRNA expression and anthocyanin levels (Fig. 4B, C), as well as between modules and anthocyanin accumulation (Fig. 4D), suggest that these modules may act as positive regulatory hubs for fruit pigmentation. By contrast, TFs negatively correlated with anthocyanin content—such as the CHS repressor *RoMYB4* (Fig. 4C)—were enriched in module 1. This module operates during ST1 (the green stage; Fig. S11), characterized by low anthocyanin levels (Fig. 4A). Its strong negative correlation with anthocyanin accumulation (Fig. 4D) suggests a potential repressive role. Notably, *MED5a*, a Mediator complex subunit known to be involved in transcriptional repression of phenylpropanoid metabolism in *Arabidopsis*^45^, is a constituent of this negatively correlated module 1 (Fig. 4D), supporting the potential robustness and biological relevance of our gene regulatory network analysis. However, without rigorous experimental validation, this pattern may simply reflect stage-specific expression differences rather than a direct inhibitory mechanism. Nevertheless, these findings suggested that a coordinated gene regulatory architecture orchestrates the distinct anthocyanin accumulation profiles during black raspberry fruit ripening.

### Non-CG methylation levels in heterochromatin in the fruit genome are higher than in leaf

**Fig. 5:**
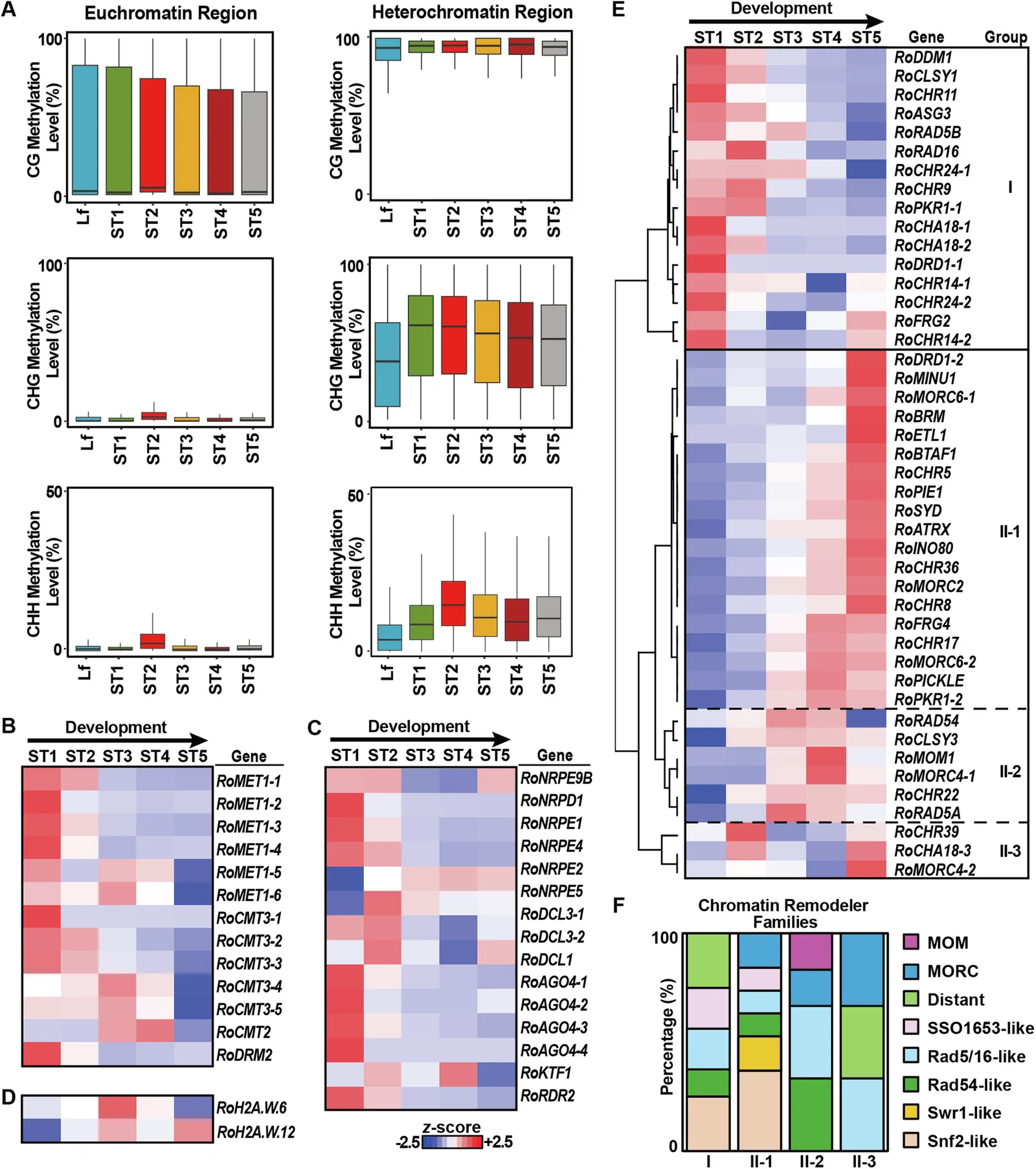
Euchromatic and heterochromatic DNA methylation and gene expression patterns of epigenetic-related genes during ripening. **A** DNA methylation levels in heterochromatic and euchromatic regions (50-bp windows) are shown as box plots. The *n* values indicate the numbers of retained 50-bp windows assigned to euchromatic or heterochromatic regions and included in each box plot after excluding windows with missing values. Lf, leaf. Heatmaps present expression of genes related to **B** DNA methylation, **C** RNA-directed DNA methylation (RdDM) pathway, **D** histone variants, and **E** chromatin remodelers. Gene expression normalized count values are shown as z-scores calculated for each gene across samples. **F** Chromatin remodeling genes are clustered into two major transcriptional groups across ripening stages by unsupervised hierarchical clustering analysis.

We uncovered hyper-DMRs are predominantly located in intergenic regions (Fig. 1J) and that the chromosomal distribution of non-CG hyper DMRs were enriched in transposon-rich heterochromatin rather than in gene-rich euchromatic regions (Fig. S4). We examined methylation levels in both euchromatic and heterochromatic regions across fruit ripening stages (Fig. S12). As expected, DNA methylation in fruit and leaf were higher in heterochromatic regions than in euchromatic regions across all three cytosine contexts. Notably, CHG and CHH methylation in heterochromatin is substantially higher in fruit compared to leaf throughout all five ripening stages—a pattern not observed in the euchromatic regions (Fig. 5A). These findings indicated that the regulation of CHG and CHH methylation in heterochromatin exhibits tissue-specific differences.

### Concurrent elevated expression of DNA methyltransferases and compaction histone genes suggests unique chromatin dynamics during ripening

To understand gene activities involved in catalyzing CHG and CHH methylation, we examined the transcript levels of DNA methyltransferase. We revealed that (i) *RoCMT2*, catalyzing CHH methylation in long transposons and heterochromatic transposons^46^, is active at the late fruit stages, suggesting that the increase in CHH methylation can, in part, be attributed to *RoCMT2*; (ii) *RoDRM2* and most RdDM pathway genes, catalyzing CHH methylation in short transposons and euchromatic transposons^46^, are predominantly expressed during early stages; and (iii) *RoCMT3*, catalyzing CHG methylation, are active from early to late ripening stages (Fig. 5B, C, and Supplementary Data 3C, D). These results suggested that a genome-wide increase in non-CG methylation within heterochromatin may be associated with elevated DNA methyltransferase transcript levels. However, heterochromatin is compact and less accessible. Therefore, increased heterochromatic DNA methylation may reflect a shift toward greater chromatin accessibility in heterochromatin, a situation resembling that observed during *Arabidopsis* embryogenesis^47–49^. Interestingly, we also found that the transcripts of constitutive heterochromatin-associated H2A.W variants^50,51^ increased during late ripening stages (Fig. 5D and Supplementary Data 2E). Thus, elevated non-CG methylation alongside increased transcript levels associated with heterochromatin may reflect a finely tuned regulatory epigenetic state that allows access to DNA methyltransferases despite the presence of compaction-associated histones. This is consistent with findings in *Arabidopsis*, where loss of H2A.W resulted in decreased accessibility and reduced non-CG methylation in pericentromeres^52^. Our finding suggests that H2A.W variants may play a role in promoting non-CG methylation during black raspberry ripening.

### Multiple chromatin remodelers are transcriptionally active during fruit ripening

To investigate mechanisms underlying elevated non-CG methylation with increased heterochromatin compaction, we analyzed the transcriptional profiles of chromatin remodelers that may facilitate heterochromatin opening. The *Arabidopsis* DDM1 is required for cytosine methylation at H1-associated heterochromatin upon cell division^46^. However, cell division ceased after the early ripening stages, and *RoDDM1* was downregulated after ST2 (Fig. 5E and Supplementary Data 3F), suggesting that additional chromatin remodelers might play roles in licensing access of methyltransferases to heterochromatin. To identify potential chromatin remodelers mediating heterochromatin accessibility after ST2, we conducted a homology-based gene search and examined their expression dynamics across fruit ripening. We identified 44 remodelers that can be clustered into two expression patterns (Fig. 5E and Supplementary Data 3F). Group I genes, including *RoDDM1*, were highly active during the early stages of fruit ripening, whereas group II genes were predominantly upregulated during later stages. For example, group II-1 consists of genes that were strongly expressed during the final stage ST5 (Fig. 5D). Interestingly, members of five gene families represented in group I also appear in group II (e.g., *RoCLSY* and *RoDRD* genes in the RoRad54-like family), suggesting a subfunctionalization to accommodate a stage-preferred function (Fig. 5D, F, and Supplementary Data 3F). Furthermore, several gene families were uniquely represented in group II, including the *RoMOM* and *RoMORC* genes (Fig. 5F). Remodelers, such as MOM1, MORCs, CLSYs, and DRDs, can influence DNA methylation or direct RdDM in a locus-specific manner^53–55^. Together, our results suggest a coordinated action of distinct chromatin remodelers active at different ripening stages to mediate chromatin accessibility and to facilitate targeted DNA methylation at specific genomic loci.

## Discussion

Dynamic methylation changes during reproduction play a critical role in reinforcing TE silencing and maintaining genome stability^56–58^. Our findings suggest that black raspberry ripening may involve similar mechanisms to ensure epigenomic integrity. To ensure comprehensive capture of DNA methylation changes during black raspberry fruit development, we identified DMRs using CGmapTools with a statistical threshold of *p* < 0.05 and minimum methylation differences of >20% for CG, >10% for CHG, and >8% for CHH. The use of this less stringent *p*-value threshold may increase the number of false-positive DMRs and could represent a limitation of this study. To mitigate this concern, we integrated the DMRs with DEGs, which further strengthened the biological support for loci showing coordinated changes in DNA methylation and gene expression during fruit development and ripening. The prevalence of intronic DMRs we observed (Fig. 1J) raises intriguing possibilities regarding post-transcriptional regulation. Intronic methylation influences alternative splicing and the generation of transcript isoforms^59^. It is tempting to speculate that the dynamic intronic methylation observed may contribute to producing transcript variants at different ripening stages. Although a detailed investigation of splicing isoforms is beyond the scope of this study, these potential developmental processes represent an exciting avenue for future research.

**Fig. 6:**
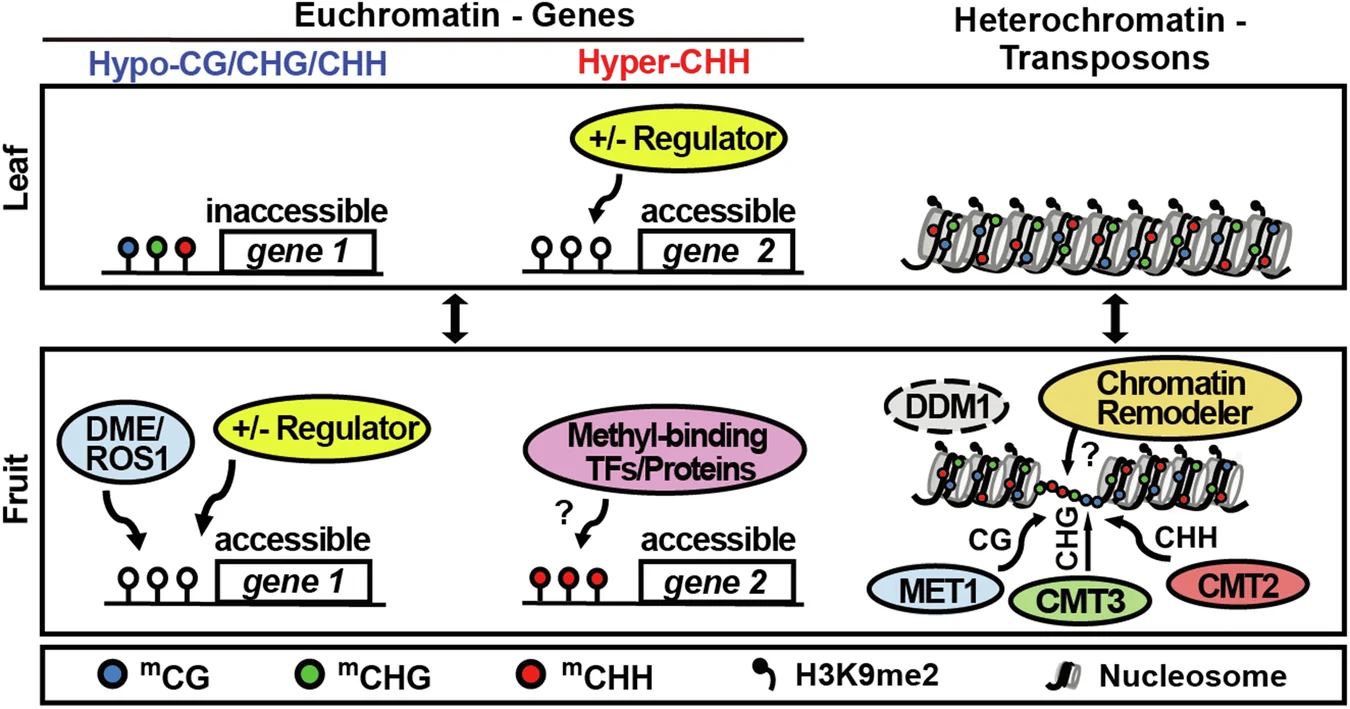
Schematic model of DNA methylation dynamics in euchromatic gene regions and heterochromatic transposons in the leaf and the fruit of black raspberry. In euchromatic gene regions, promoter-localized CG, CHG, and CHH demethylation, mediated by DME/ROS1 DNA demethylase, is associated with transcriptional regulation, whereas CHH hypermethylation is associated with transcriptional activation or repression at specific loci together with methyl-binding proteins and TFs. In heterochromatic transposons, elevated non-CG methylation is accompanied by dynamic expression of chromatin remodelers, which may facilitate methyltransferase access during fruit development.

We observed that localized CG demethylation occurs primarily at promoter regions where CHG and CHH are concurrently demethylated (Fig. 6). Promoter methylation changes during fruit development are associated with altered transcription and are enriched for fruit ripening functions. DNA demethylases act at specific loci to regulate gene activity^31^. Yet, how the demethylases are recruited to target loci remains largely unknown. The tomato *SlDML2* is highly induced upon the onset of ripening^6,7^. CRISPR knockout of *SlDML2* delayed fruit ripening and led to DNA hypermethylation in thousands of genes, including ripening-induced and repressed genes^6^, consistent with our finding that CG/CHG/CHH hypo-DMRs-associated genes include up- and down-regulated DEGs in black raspberry (Fig. 2C). Tomato genes with ripening-induced hypo-DMRs include *NOR*, *RIN*, *CNR*, and *DML2*, indicating their expression is regulated by DNA demethylation. Moreover, *NOR* directly targets and activates the transcription of *SlDML2*, forming a positive feedback loop to sustain elevated *SlDML2* and *SlNOR* expression during tomato ripening^32^. This is also consistent with the high expression of *RoDML3* and *RoNOR* throughout fruit development (Fig. 1I and Fig. S9B, D). The *Cnr* CRISPR mutants delayed ripening, while *nor* CRISPR lines showed partial non-ripening phenotypes, which were different from the strong non-ripening phenotypes of their natural mutants^34–36^, indicating that the natural mutants were not simple loss-of-function alleles. The high *RoNOR* expression during ripening is accompanied by promoter CG demethylation as the fruit ripens (Fig. S9B). The promoter demethylation and transcript accumulation of *RoNOR* are independently validated by Chop-PCR and qRT-PCR assays (Fig. S9C, D). In contrast to what was observed in tomato, *RoCNR*, *RoTDR4*, and *RoRIN* all show strong transcription repression as ripening progresses (Fig. S9D). This is likely attributed to the differences between climacteric and non-climacteric fruits^60,61^. The tomato TDR4, FUL2, TAGL1, and RIN are known to form a complex that binds to the promoters and regulates the expression of ethylene biosynthesis genes during tomato fruit ripening^62^, which might function differently in black raspberry.

We observe that CHH hypermethylation is linked to gene activation and repression during black raspberry fruit ripening (Fig. 6), in agreement with studies in strawberry^20^. DNA methylation is intimately linked to transcriptional repression by impeding TF binding, recruiting repressor complexes, or by compacting local chromatin to restrict transcription^11^. Promoter methylation can also contribute to active gene transcription. The promoter of the *ROS1* gene is methylated by RdDM for active transcription^12,13^. Together, these studies highlight how sequence-specific TFs control the spatial and temporal regulation of DNA methylation and provide a genetic framework for elucidating epigenetic patterning and transcriptional regulation in fruit ripening.

Black raspberry fruits show substantially elevated non-CG methylation in heterochromatin, indicating a fruit-specific heterochromatin remodeling program and is reminiscent of the *Arabidopsis* endosperm, pollen, and embryo^47–49,58,63^. We observed that many chromatin remodeler genes are dynamically regulated at transcriptional level at distinct stages, which may contribute to the elevated non-CG methylation (Fig. 6). For example, *RoDDM1*, *RoCLSY1*, *RoDRD1* are highly active during early stages (Fig. 5E) and may facilitate chromatin accessibility to the RdDM machinery whereas remodelers active during later ripening stages like *RoBRM*, *RoPIE1*, *RoSYD*, *RoINO80*, and *RoPICKLE* may contribute to exchanging histone variants, altering nucleosome positioning, or epigenetically regulating gene transcription. Our results depict a dynamic, tightly regulated, and concerted molecular framework whereby epigenomic modifications, transcriptional reprogramming, chromatin remodeling, and metabolic shifts act in concert to orchestrate fruit ripening processes in black raspberry.

## Methods

### Plant material

Black raspberry NC 349, an advanced selection from a Jewell x NC 100 cross developed by Dr. James Ballington, was planted at the North Carolina Department of Agriculture Piedmont Research Station in Salisbury, NC in 2012. Fruits and leaves used in this study were collected between June 10 and July 20, 2016. Black raspberry fruit development progresses through distinct color and firmness changes, from green to red, then violet, and finally black. Black raspberry leaves and fruits at five ripening stages were collected (Fig. 1A), and bio-replicates of each were collected to make RNA-seq libraries, BS-seq libraries, and for the metabolome experiments.

### Reference genome used in this study

The reference genome of *Rubus occidentalis* v3.0 was downloaded from Genome Database for Rosaceae (GDR, https://www.rosaceae.org/analysis/268), and used for mapping of sequencing reads, data processing, and subsequent analysis. The genome assembly and annotation of black raspberry have been previously reported^28,64,65^.

### Genomic DNA and total RNA isolation

Three bio-replicates of black raspberry fruits were collected from multiple trees at the five ripening stages. Collected fruits were flash-frozen in liquid nitrogen in the field and later stored at −80 °C. Approximately 4–6 fruits from each stage were ground into fine powder using a mortar and pestle under liquid nitrogen. The ground powder from each sample was separated into two parts for subsequent genomic DNA and total RNA isolation. Genomic DNA was isolated using the DNeasy Plant Mini Kit (Qiagen) according to the manufacturer’s instructions. Total RNA was isolated using the Spectrum Plant Total RNA kit (Sigma) following the manufacturer’s protocol. Due to the high content of phenolic compounds in the fruits, the total RNA was further purified by polyethylene glycol (PEG 35,000; Fluka) and cleaned up using the RNeasy Plant Mini Kit (Qiagen) according to Gehrig et al.^66^.

### BS-Seq library construction, methylome sequencing, data processing, and sequence analysis

Fruits of five ripening stages (Fig. 1A) with two bio-replicates were collected to extract genomic DNA as subject for sodium bisulfite treatment followed by BS-seq library construction. In addition, genomic DNA extracted from leaves was also used for BS-seq library construction as a control to compare methylation levels with fruits. These BS-seq libraries were sequenced using the Illumina platform HiSeq X ten with 150-base paired-end reads. DNA was prepared for BS-Seq library preparation and methylome sequencing following the methods of previous published methods^57^. Specifically, DNA sequences were aligned to the black raspberry whole genome assembly and annotation (version 3.0; https://www.rosaceae.org/analysis/268)^28,64,65^. Adapter-ligated DNA libraries underwent two rounds of BS treatment using the EZ DNA Methylation-Lightning Kit (Zymo Research). Following BS conversion, DNA was purified and amplified for 10 cycles using Q5U DNA polymerase (NEB). Size selection of PCR-amplified fragments was performed using AMPure XP beads (Beckman). Sequencing reads were aligned to the reference genome using BS-Seeker2^67^, allowing up to four mismatches. Cytosine coverage was calculated as the total number of cytosines (methylated) and thymines (converted/unmethylated) detected across all reads. Only uniquely mapped reads were retained for downstream analysis. Postprocessing of the BS-Seeker2 output involved two quality control steps: (i) removal of PCR duplicates by collapsing reads with identical 5′-end mapping positions and nucleotide sequences, and (ii) filtering out reads containing three or more consecutive cytosines in the CHH context, which are indicative of incomplete BS conversion^57^.

### Assessment of bisulfite conversion efficiency

To evaluate the efficiency of bisulfite (BS) conversion and estimate the non-conversion rate, we included 3 ng of unmethylated cI857 Sam7 λ DNA (GenBank Accession No. NC_001416; Promega) as an internal control by spiking it into each genomic DNA sample prior to sonication for library preparation. Sequencing yielded a minimum of 300× coverage across the λ genome, allowing for robust analysis. We detected more than 99% of C-to-T conversion within the λ genome, indicating highly efficient BS conversion.

### DMR analysis by CGmapTools

To identify the changes in local DNA methylation levels, which is called differentially methylated regions (DMRs), during black raspberry fruit ripening, we applied CGmapTools^29^ to identify DMRs. The analysis was performed using the following parameters: a minimum of five common cytosine sites per DMR, a maximum distance of 100 bp between adjacent sites, a read coverage range of 2–500, and a maximum fragment size of 200 kb. For each paired sample between any two stages of the five developmental stages, only cytosine sites covered by sufficient reads (≥2×) in both samples were retained for DMR identification. Additionally, a maximum coverage cutoff of 500 was applied for DMR calculation. Moreover, cytosine sites showing sequence mismatches between the BS-seq reads and the reference genome were excluded from all downstream analyses to avoid false-positive methylation calls. CGmapTools reports statistical test results and corresponding unadjusted *p* values for all candidate fragments and does not apply an additional multiple-testing correction by default. Candidate fragments were subsequently defined as DMRs using a significance threshold of *p* < 0.05 together with context-specific methylation-difference thresholds of ΔCG > 20%, ΔCHG > 10%, and ΔCHH > 8%. Adjacent DMRs separated by less than 300 bps were merged using the merge function implemented in BEDTools, following the <300-bp criterion reported previously^58^. To precisely and comprehensively inspect the occurrence of DMRs, DNA methylation levels in leaves were used as a baseline reference for comparison with those in each fruit ripening stage.

### Identification of differentially methylated genes by colocalization analysis

We defined differentially methylated genes (DMGs) as genes that have differentially methylated regions (DMRs) in the promoter (1.5 kb upstream of the transcription start site) and gene body. We scanned the genome using the intersect function of BEDTools^68^ with coordinates from DMRs and all genes in the black raspberry genome to colocalize DMRs and genes.

### RNA-Seq library construction, sequencing, data processing, and analysis

Three bio-replicates each of black raspberry fruits were collected at the five ripening stages. Total RNA was isolated from fruits and leaves using the RNeasy Plant Mini Kit (Qiagen) and treated with RNase-free DNase I (Qiagen). Total RNA with a minimum RIN (RNA Integrity Number) value of 8 was employed for RNA-Seq library construction. Poly-A+ RNA was selected using the NEBNext Poly(A) mRNA Magnetic Isolation Module (NEB) for RNA-Seq library construction using the KAPA Stranded mRNA-Seq Kit (Roche).

RNA-seq libraries were sequenced using the Illumina platform HiSeq 4000 to generate 150-base paired-end reads. Previously published methods were applied to process RNA-Seq data with modifications^57^. Specifically, Illumina raw reads were processed with Trimmomatic^69^ to eliminate adapters and to trim low-quality bases at the 5’ and 3’ ends, targeting positions with error rates greater than 0.5% and error rate > 0.1%. The remaining high-quality reads were mapped to the reference genome using HISAT2^70^ with mismatch 4. Only uniquely mapped reads (i.e., reads that map to one unique genomic locus) were used for subsequent analysis. The read counts per gene were analyzed using HTSeq^71^, followed by using DESeq2^72^ to determine DEGs with cutoff *q* < 0.05 and > two-fold changes. Normalized counts generated by DESeq2 were used for downstream gene expression analyses. Non-supervised hierarchical clustering was analyzed by dChip^73^. To generate a comprehensive Gene Ontology (GO) database for black raspberry, we used the NCBI non-redundant protein database and Blast2GO^74^ with BLASTP parameters: an *E*-value cutoff of 1 × 10^−4^ was applied to ensure the stringency of the sequence alignment. The annotation was conducted using the default parameters for mapping and annotation steps, consistent with the specified BLASTP filters. This approach enabled the assignment of functional GO terms to the predicted protein-coding genes to construct a GO database. The goseq package^75^ was used for gene GO enrichment analysis with a cutoff FDR (Benjamini–Hochberg multiple testing correction) < 0.05.

### Identification of genes whose transcriptional activity correlates with alterations in DNA methylation

DMGs and differentially expressed genes (DEGs) were integrated to investigate the relationship between DNA methylation and gene expression. Genes that were both differentially methylated and differentially expressed were identified as genes whose transcriptional activity is regulated by DNA methylation.

### Metabolome profiling

Three biological replicates of fruits at the five ripening stages (Fig. 1A) were collected to extract metabolites for high-performance liquid chromatography with diode-array detection (HPLC-DAD), following previously published methods^76^. Specifically, filtered samples were injected (10 µL) into a 1200 HPLC system (Agilent Technologies, Santa Clara, CA, USA) equipped with a UV–vis diode array detector (DAD), controlled-temperature autosampler (4 °C), and column compartment (30 °C) using a reversed-phase Supelcosil LC-18 column, 25 mm × 4.6 mm × 5 µm (Supelco, Bellefonte, PA, USA). Standard curves were calculated using peak areas at UV of 520 nm as cyanidin-3-O-glucoside equivalents.

### Homology search to deduce enzyme-coding genes in anthocyanin biosynthesis

With respect to the metabolite profiles and the addition of functional groups to the substrates, we predicted enzymes that catalyze the transfer reaction to produce the seven anthocyanins (Fig. S10), including anthocyanidin 3-O-glucoside 2-O-xylosyltransferase, anthocyanin 3-O-glucoside rhamnosyltransferase, anthocyanidin 3-O-glucoside xylosyltransferase, anthocyanidin 3-O-glucosyltransferase and anthocyanin O-methyltransferase. We conducted a protein homology search using protein sequences from other plant species with experimentally proven enzyme functions to BLASTP (*E* < 10^−4^) against the black raspberry protein database to identify candidate genes with the most significant *E*-values in the black raspberry genome.

### Identification of anthocyanin-, ripening-related and methyl-binding transcription factors in black raspberry

To identify putative transcription factors (TFs) involved in fruit ripening and anthocyanin biosynthesis in black raspberry, we first searched publications and review articles related to these processes across various plant species (Supplementary Data 10). According to these previous studies, we selected TFs that were either functionally validated or widely recognized in the literature. Subsequently, the protein sequences of these known TFs were retrieved and subjected to BLASTP analysis against the protein database of black raspberry. For each query sequence, the hit with the lowest *E*-value (threshold: 1 × 10⁻⁴) was selected as the best match. In cases where multiple query TFs aligned to the same black raspberry TF, we retained the one with the most significant *E*-value. If two or more hits had identical *E*-values, the TF from the species phylogenetically closest to black raspberry or with the highest protein sequence identity was selected as the representative. This approach ensured that each well-characterized TF from other species was systematically assigned to a corresponding blackberry homolog with the highest confidence.

Candidate black raspberry DNA methyl-binding transcription factors were identified based on homology to well-characterized *Arabidopsis* orthologs using BLASTP (*E* < 10^−4^). This initial list was filtered to include only those members with experimental evidence for DNA methylation-related transcriptional regulation in published literature. The final selection, including black raspberry homologs, transcript level, and corresponding references, is documented in Supplementary Data 3B.

### Weighted gene correlation network analysis (WGCNA)

The WGCNA R package^77^ was employed to identify gene coexpression modules of highly correlated expressed genes using DESeq2 normalized data, following the framework in Wang et al.^78^ with modifications as described below. Specifically, we first plotted the distribution of RNA abundance for each ripening stage using a density plot based on log₂(DESeq2 normalized count + 1) values. We identified the lowest point between the two peaks of the bimodal distribution, which represents the threshold for low expression. Genes with expression levels below this point were considered to be expressed at low levels and excluded from the WGCNA analysis. The lowest points for the five ripening stages (from green to black) were 3.06, 3.49, 3.12, 3.16, and 3.91, respectively. Finally, the lowest value (3.06) among the five stages was used as the cutoff across all stages.

For WGCNA construction, an appropriate soft-thresholding power was selected to ensure that the resulting network approximated a scale-free topology. Network topology analysis was conducted across the first 30 candidate powers, and a soft threshold of *β* = 20 was ultimately chosen. This threshold yielded a slightly relaxed scale-free topology fit index (*R*² > 0.7), which is acceptable given the limited sample size (*n* = 14, excluding one ST4 outlier as described in the Results section) and reflects the constraints of the experimental system^79^. Based on pairwise gene-to-gene correlations, an adjacency matrix was constructed using the selected power and subsequently transformed into a topological overlap matrix (TOM) to quantify gene co-expression similarity. Gene modules were identified using the dynamic tree-cutting algorithm (deepSplit = 2, minClusterSize = 30), and highly similar modules were merged using a cut height threshold of MEDissThres = 0.2. This procedure resulted in the identification of robust and reliable gene modules with clear co-expression structure and minimal noise^80^. To evaluate module robustness, we performed a permutation test demonstrating that the average topological overlap within identified modules was significantly higher than that observed in randomly sampled gene sets (*p* < 10⁻⁵). These results support the reliability of the constructed gene regulatory modules.

### Candidate gene validation

Genomic DNA from leaves and five stages of fruit samples were digested with methylation-sensitive restriction enzymes BstBI (for CG context), AluI (for CHH context), or McrBC (for all contexts) (New England Biolabs)^81^ for Chop-PCR. A duplicated set of reactions were used as no-digestion control. Approximately 3–5 ng of digested and undigested control DNA were used for PCR amplification (30 cycles). For qRT-PCR, 0.5 μg of total RNA was reverse-transcribed using the LunaScript® RT SuperMix Kit (New England Biolabs, Inc.). The cDNA was diluted fivefold with H_2_O. For each 20-μL qPCR reaction, 1 μL (approximately 3–4 ng) of diluted cDNA was used along with the Luna® Universal qPCR Master Mix on an Applied Biosystems 7500 Fast Real-Time PCR System. Ct values were normalized against the RO-ACT mRNA. The abundance of mRNA was expressed relative to ST1, with the control value set to 1. Error bars represent the standard deviation of four biological replicates. The Chop-PCR and qRT-PCR primer sequences are listed in Supplementary Data 11.

### Sampling heterochromatic and euchromatic regions of black raspberry genome

The euchromatin and heterochromatin regions were randomly selected through DNA methylation profiling, transposon, and gene density across seven chromosomes of black raspberry by Integrative Genomics Viewer v.2.19.4^82^. The region with a high level of DNA methylation, high transposon density, and low gene density was defined as the heterochromatin region. Conversely, the region with a low level of DNA methylation, low transposon density and high gene density was defined as the euchromatin region.

### Statistics and reproducibility

For each cytosine in the reference genome, we quantified the number of reads reporting cytosines (methylated cytosines) and thymines (resulting from bisulfite conversion of unmethylated cytosines), using custom scripts. Sites with only a single mapped read (either cytosine or thymine) were excluded to reduce noise. To further minimize false positives, we applied a binomial test (*p* < 0.05) to determine whether the observed methylation at a site was statistically significant. Cytosines passing this threshold—typically those with at least two cytosine reads—were considered methylated. Conversely, cytosine sites with at least one thymine read and failing the binomial test were considered unmethylated, indicating that bisulfite conversion of unmethylated cytosines to uracils (detected as thymines in sequencing). DNA methylation levels were determined for CG, CHG, and CHH contexts following the terminology in Schultz et al.^83^. For window-based analyses, the genome was scanned using 50-bp window, and weighted methylation levels were calculated by using the formula: [*C*/(*C* + *T*)] × 100, where *C* represents the total number of methylated reads and *T* represents the total number of unmethylated reads within each window. The mean methylation level method was applied for calculation of the average methylation level of a single cytosine site in the genome.

To explore the global DNA methylation differences of leaf stage and five fruit ripening stages, the two-sided Wilcoxon test with FDR correction was used. The associations between gene modules and phenotypic traits (metabolite data), Pearson’s correlation was calculated between module eigengenes and sample traits, and the significance of each correlation was assessed by a two-sided Student’s *t* test.

To assess the consistency of biological replicates of BS-Seq data, two biological replicates were collected for each of the five fruit ripening stages and the leaf stage (*n* = 12). Pearson’s correlation coefficient was calculated using methylation levels in 10-kb windows and visualized in a heat map using the “pheatmap” package in R. Besides, PCA was performed on the average methylation levels in 10-kb windows with cytosine site ≥5× coverage across the genome for three contexts. The methylation level data were standardized to mean = 0 and standard deviation = 1 across 12 biological replicates. For RNA-Seq, three biological replicates were collected for each of the five fruit ripening stages; one ST4 sample was excluded as an outlier, resulting in 14 samples used for downstream analysis. For the reproducibility of biological replicates of RNA-Seq data, PCA was performed on transcript data transformation by (log10[x + 1]) from five fruit ripening stages. For metabolome profiling, three biological replicates were collected for each of the five fruit ripening stages (*n* = 15).

## Supplementary information

Supplementary Information

Supplementary Data 1

Supplementary Data 2

Supplementary Data 3

Supplementary Data 4

Supplementary Data 5

Supplementary Data 6

Supplementary Data 7

Supplementary Data 8

Supplementary Data 9

Supplementary Data 10

Supplementary Data 11

## Data Availability

The raw and processed data reported in this paper have been deposited in the Gene Expression Omnibus (GEO) database. Accession number GSE311119 for the methylome and GSE311426 for the transcriptome. Genome-wide processed datasets, the 50-bp window DNA methylation values used for downstream analyses, are available as processed data files in the corresponding GEO records. The metabolomics data have been deposited to MetaboLights^84^ repository with the study identifier MTBLS15819. The source data for the corresponding main figures are provided in the relevant Supplementary Data, as indicated in their captions. The Supplementary Data tables are provided as Supplementary Data 1–11.

## Code availability

All relevant scripts used for data processing and analysis in this study are publicly available in the GitHub repository (https://github.com/WeihsunHsieh/Ro_methylome_study) under the MIT License, and have been permanently archived and released in Zenodo (https://doi.org/10.5281/zenodo.21485750)^85^. The repository contains the scripts and associated files required to reproduce the data processing and analysis workflows described in this study.
