# Supplementary material for: Multi-omics analyses reveal dynamic interactions between DNA methylation and transcriptional regulation during black raspberry ripening
Source: Commun Biol. 2026 Nov 12;9:1463. doi: 10.1038/s42003-026-11289-3 (PMC13759512; doi:10.1038/s42003-026-11289-3)
Supplement: Supplementary file 1 [file 42003_2026_11289_MOESM1_ESM.pdf]

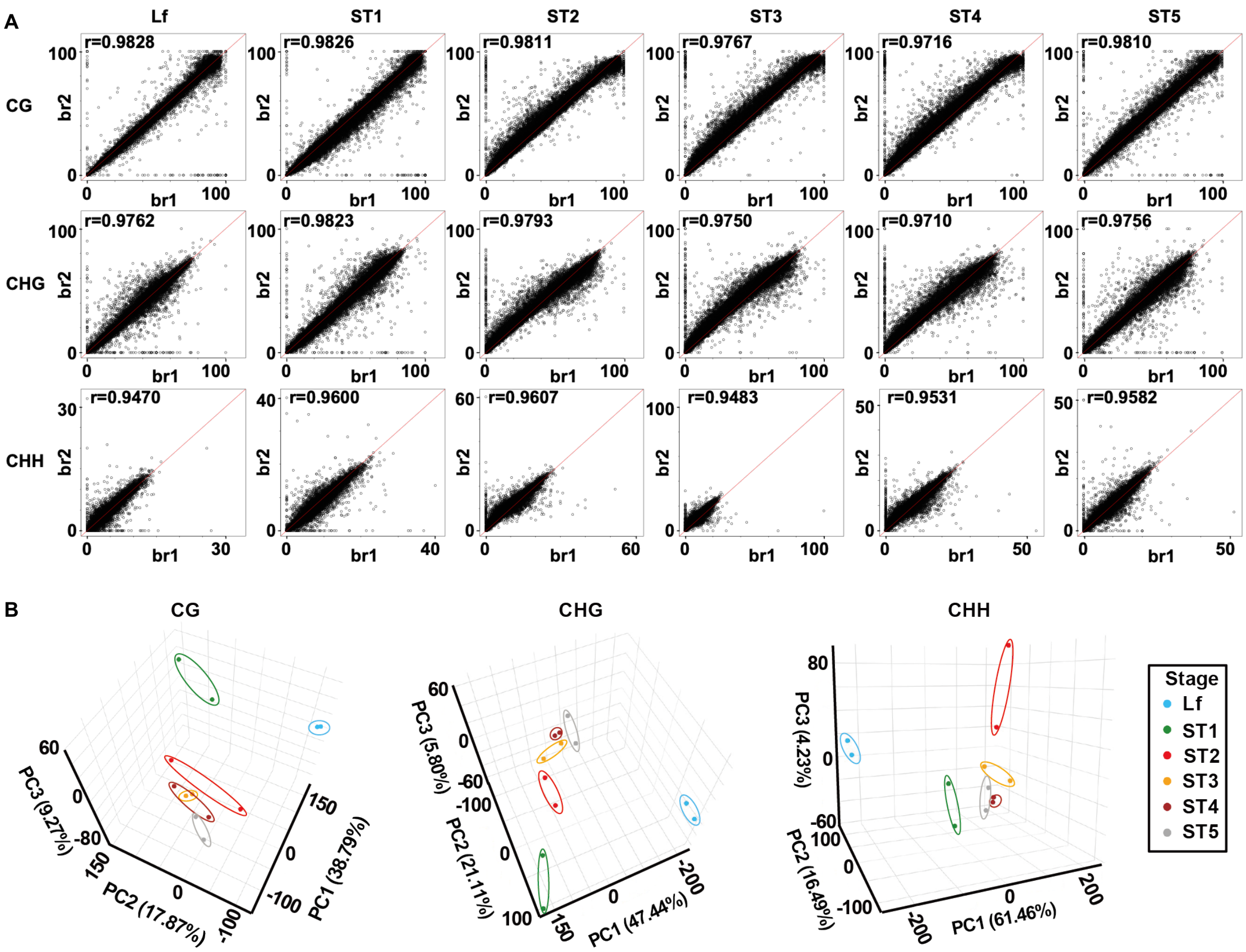

Supplementary Figure 1 | Quality assessment of BS-Seq methylome libraries. A Scatter plots of Pearson correlation coefficients between biological replicates. **B** Context-specific PCA performed using the standardized 10-kb methylation levels across 12 biological replicates.

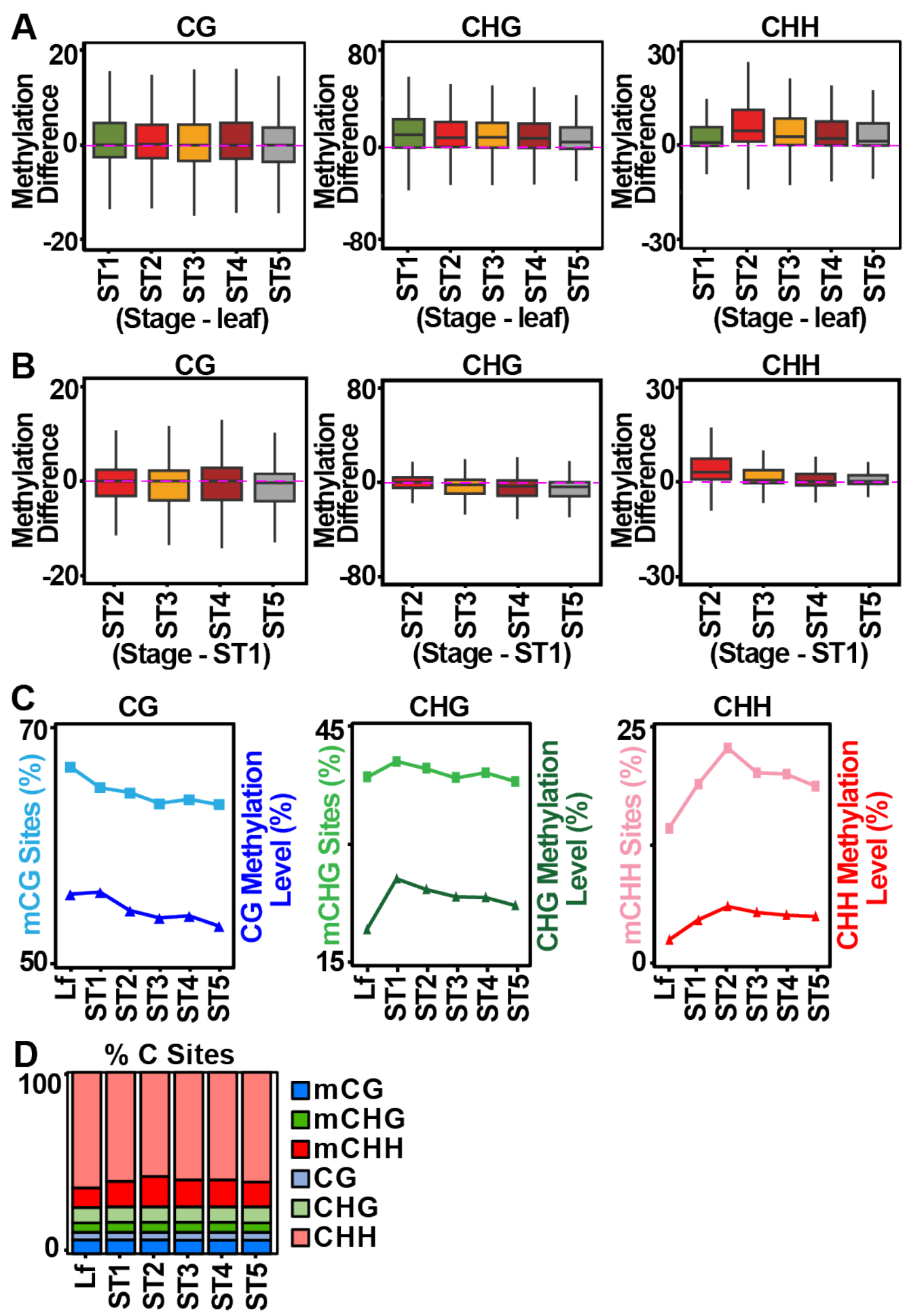

Supplementary Figure 2 | DNA methylation dynamics during black raspberry fruit ripening. A Genome-wide DNA methylation levels in fruit versus leaf tissues analyzed in 50-bp windows. **B** Differential DNA methylation between late ripening stages versus stage 1 calculated using 50-bp windows. Significant test was applied by two-sided Wilcoxon test with FDR correction in Supplementary Data 2 (*FDR <0.05 and fold change >1.2x). **C** DNA methylation changes during black raspberry fruit development assessed by comparing the proportion of methylated cytosine sites and the average methylation percentages. The latter represents the overall mean DNA methylation levels across all cytosine sites in the black raspberry genome. **D** Proportions of cytosine bases in CG, CHG, and CHH contexts for the black raspberry fruit genome. Lf, leaf; ST, stage.

A

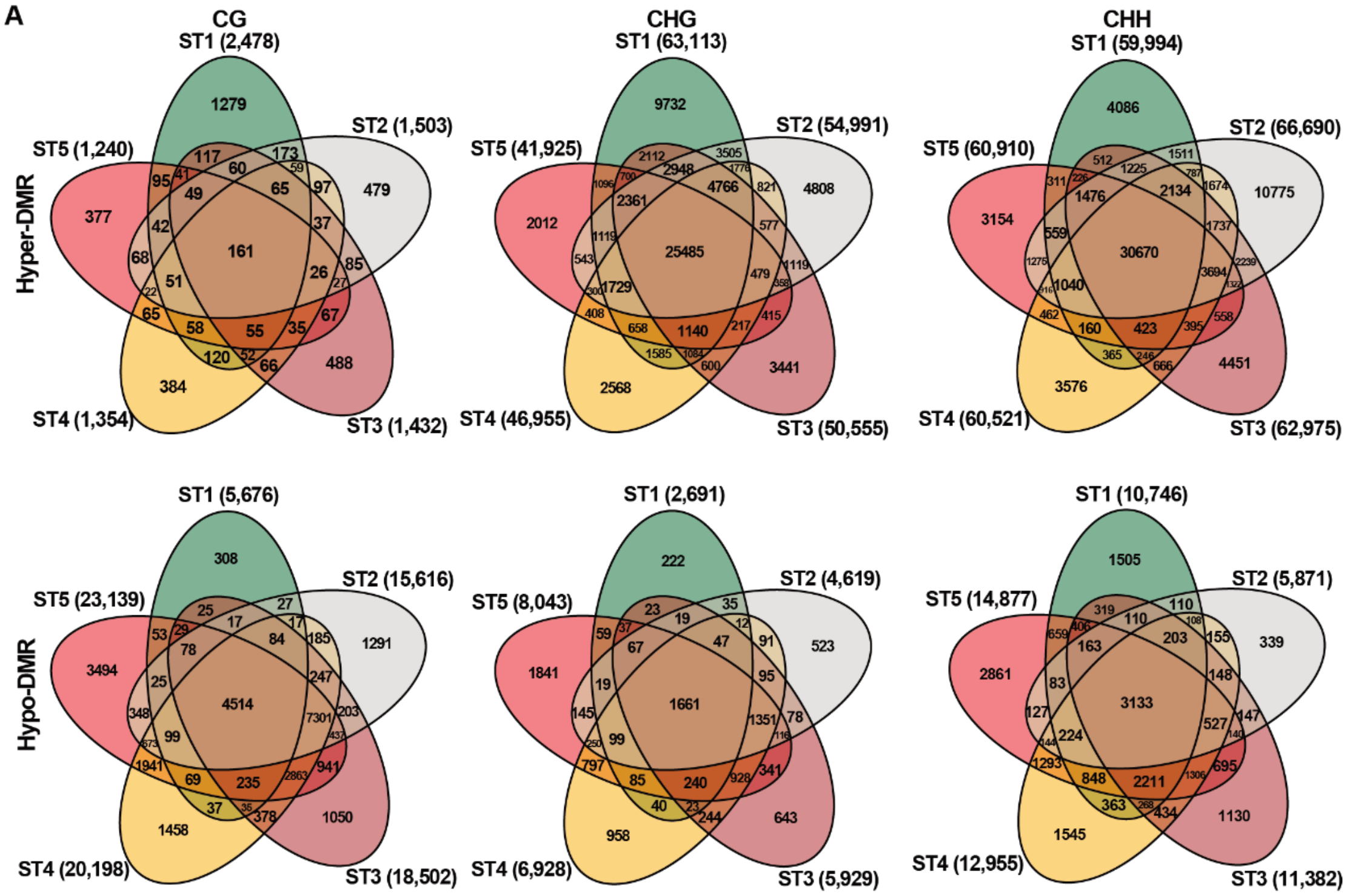

B

	CG-hyper-DMR				CHG-hyper-DMR				CHH-hyper-DMR			
Stage	Non-unique hyper DMR	Unique hyper DMR	Percentage of overlap	Total	Non-unique hyper DMR	Unique hyper DMR	Percentage of overlap	Total	Non-unique hyper DMR	Unique hyper DMR	Percentage of overlap	Total
ST1	1,199	1,279 (51.6%)	6.5%	2,478	53,381	9,732 (15.4%)	40.6%	63,113	55,908	4,086 (6.8%)	53.3%	59,994
ST2	1,024	479 (31.9%)	10.7%	1,503	50,183	4,808 (8.7%)	46.6%	54,991	55,915	10,775 (16.2%)	47.9%	66,690
ST3	944	488 (34.1%)	11.2%	1,432	47,114	3,441 (6.8%)	50.6%	50,555	58,524	4,451 (7.1%)	50.7%	62,975
ST4	970	384 (28.4%)	11.9%	1,354	44,387	2,568 (5.5%)	54.5%	46,955	56,945	3,576 (5.9%)	52.8%	60,521
ST5	863	377 (30.4%)	13.0%	1,240	39,913	2,012 (4.8%)	61.1%	41,925	57,756	3,154 (5.2%)	52.5%	60,910
Overlap	161				25,603				31,951			
	CG-hypo-DMR				CHG-hypo-DMR				CHH-hypo-DMR			
Stage	Non-unique hypo DMR	Unique hypo DMR	Percentage of overlap	Total	Non-unique hypo DMR	Unique hypo DMR	Percentage of overlap	Total	Non-unique hypo DMR	Unique hypo DMR	Percentage of overlap	Total
ST1	5,368	308 (5.4%)	79.5%	5,676	2,469	222 (8.2%)	61.7%	2,691	9,241	1,505 (14.0%)	29.2%	10,746
ST2	14,325	1,291 (8.3%)	28.9%	15,616	4,096	523 (11.3%)	36.0%	4,619	5,532	339 (5.8%)	53.4%	5,871
ST3	17,452	1,050 (5.7%)	24.4%	18,502	5,286	643 (10.8%)	28.0%	5,929	10,252	1,130 (9.9%)	27.5%	11,382
ST4	18,740	1,458 (7.2%)	22.3%	20,198	5,970	958 (13.8%)	24.0%	6,928	11,410	1,545 (11.9%)	24.2%	12,955
ST5	19,645	3,494 (15.1%)	19.5%	23,139	6,202	1,841 (22.9%)	20.7%	8,043	12,016	2,861 (19.2%)	21.1%	14,877
Overlap	4,514				1,661				3,133			

Supplementary Figure 3 | Overlap of differentially methylated regions (DMRs) among black raspberry fruit ripening stages using leaf tissues as the reference. A The number of DMRs shown as Venn diagrams. **B** The number and percentage of DMRs that are unique to a single stage or shared across more than one stage (non-unique).

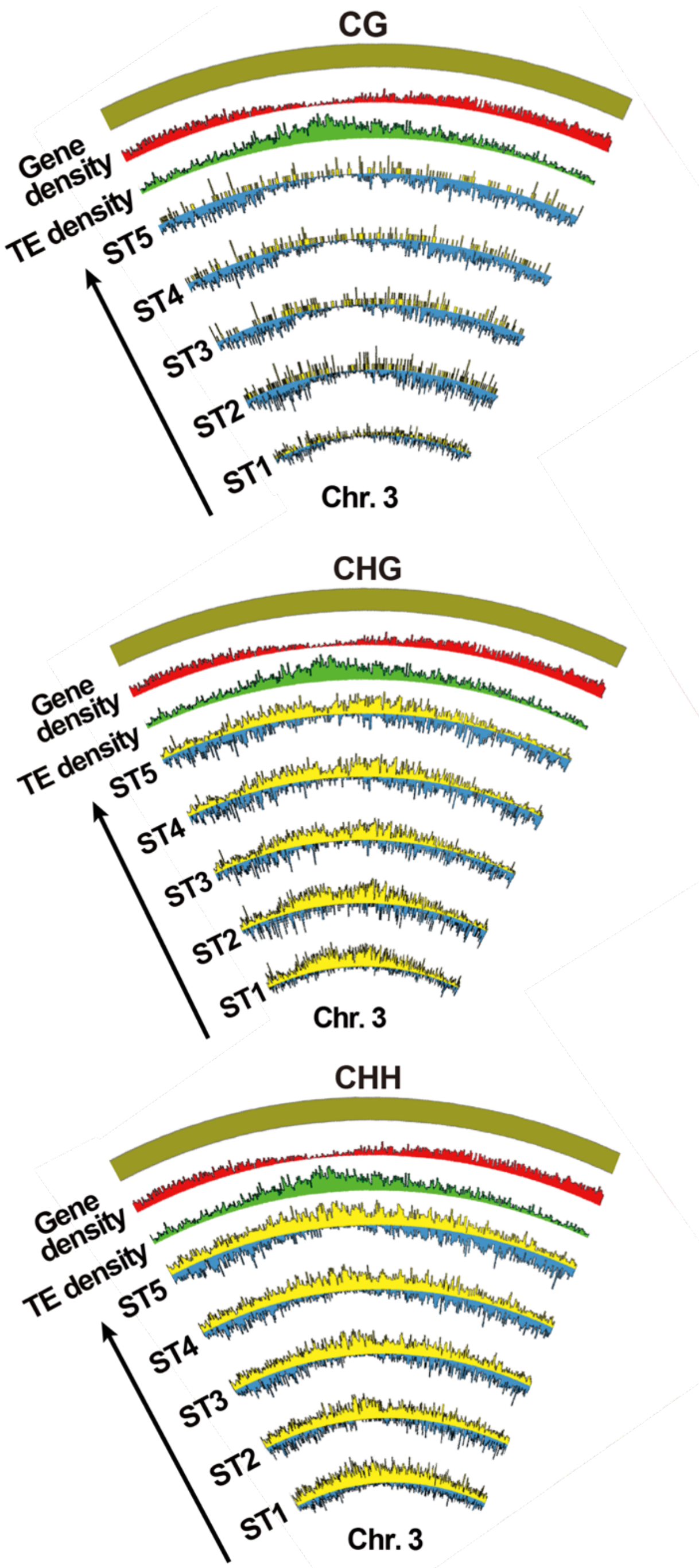

Supplementary Figure 4 | Local DNA methylation changes during black raspberry fruit ripening. The distribution of differentially methylated regions (DMRs) across chromosome 3, using leaf as the reference, is shown as an example. Hypo-methylated DMRs are shown in blue, and hyper-methylated DMRs are shown in yellow. Gene (red) and transposable element (TE; green) tracks represent the densities of genes and LTR retrotransposons along the chromosome.

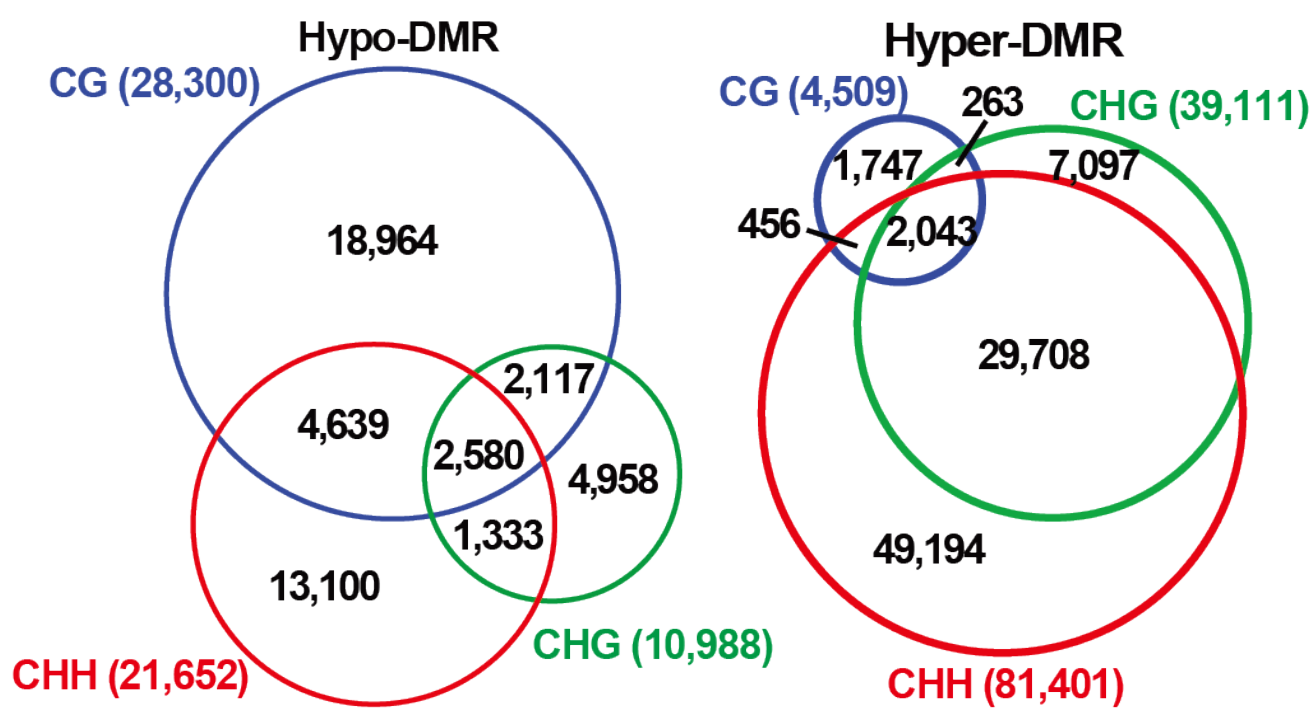

Supplementary Figure 5 | Overlap of differentially methylated regions (DMRs) across CG, CHG, and CHH contexts using leaf as reference.

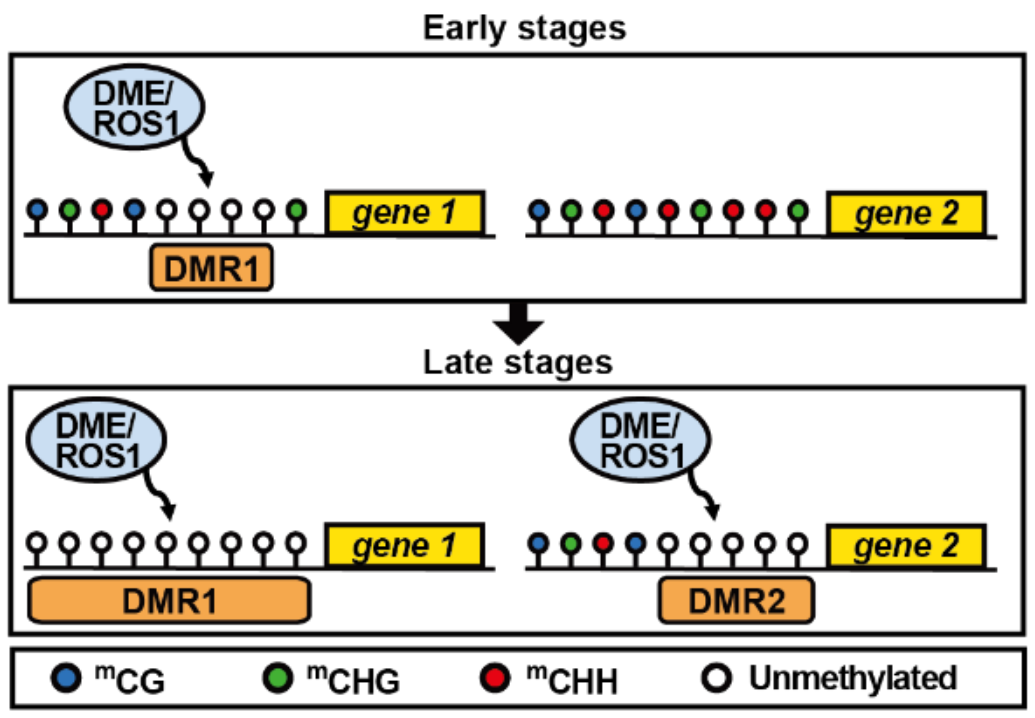

Supplementary Figure 6 | Illustration of the spatial and temporal progression of DNA demethylation during fruit ripening. Specific loci are demethylated at the early stages and gradually expand, while others maintain methylation until later stages.

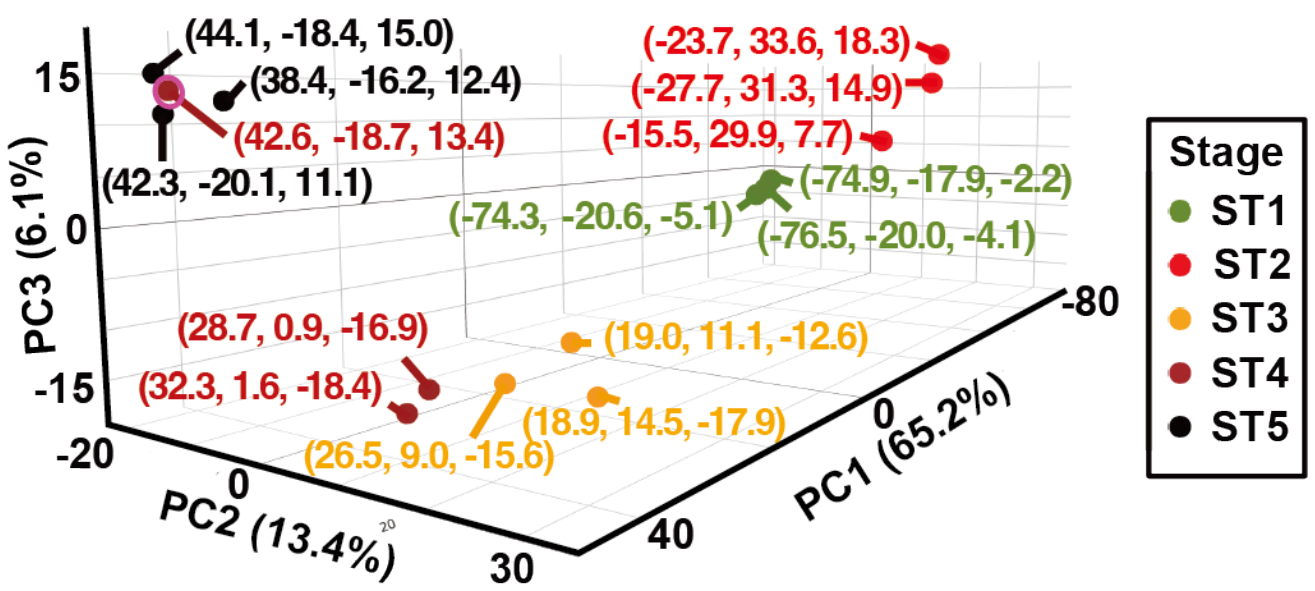

Supplementary Figure 7 | Genome-wide gene expression patterns during black raspberry fruit ripening. Principal Component Analysis (PCA) was performed on transcript data from different fruit stages. Principal Components 1 through 3 (PC1–PC3) together accounted for 85% of the variance in mRNA expression across the five stages. The circled outlier of ST4 (42.6, -18.7, 13.4) was excluded for further DEG analysis.

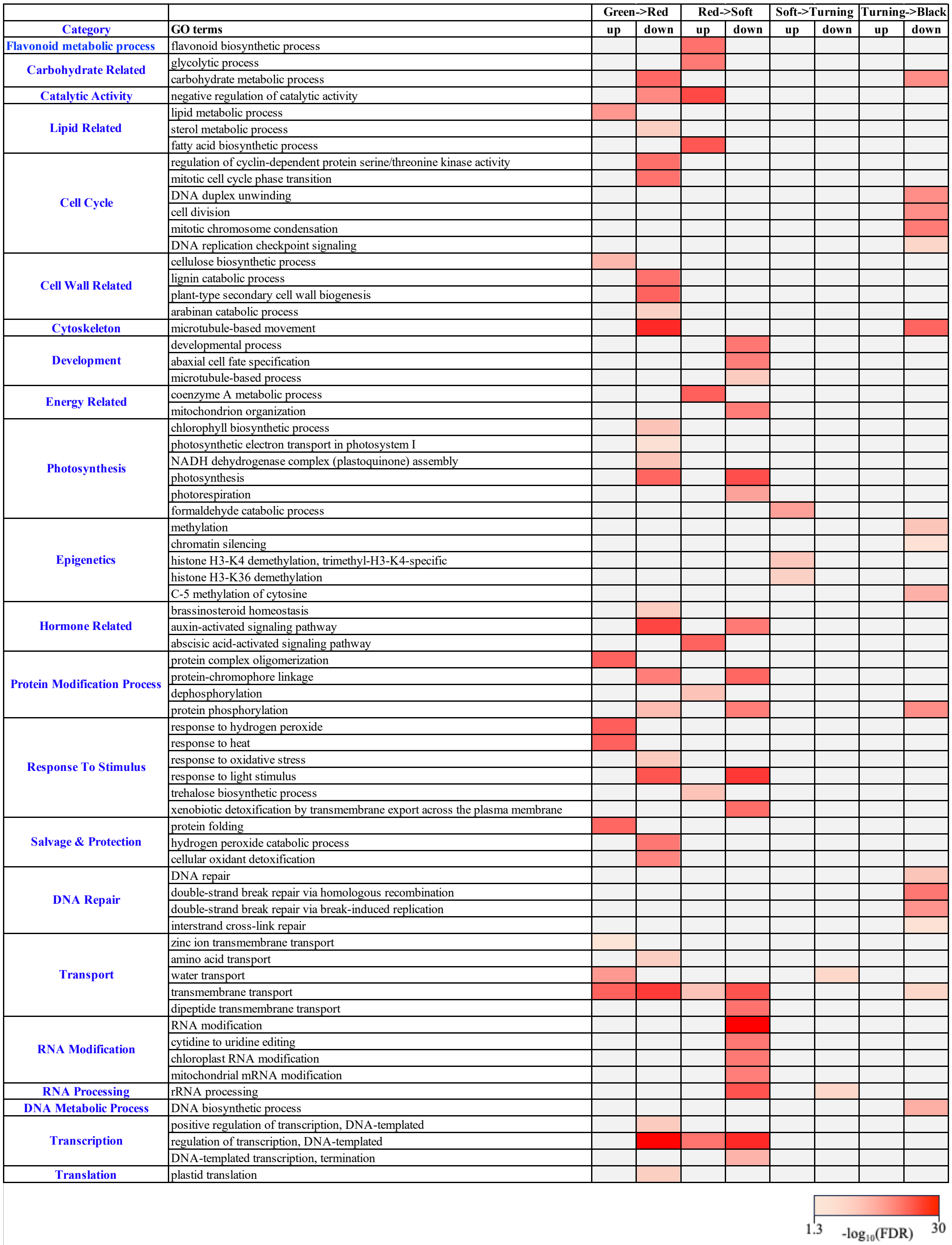

Supplementary Figure 8 | Gene Ontology (GO) analysis of differentially expressed genes (DEGs) between consecutive stages of black raspberry fruit ripening. Representative enriched GO terms

are shown, and the full list is available in Supplementary Data 5. “Up” and “Down” indicate genes that are upregulated or downregulated, respectively, between adjacent ripening stages.

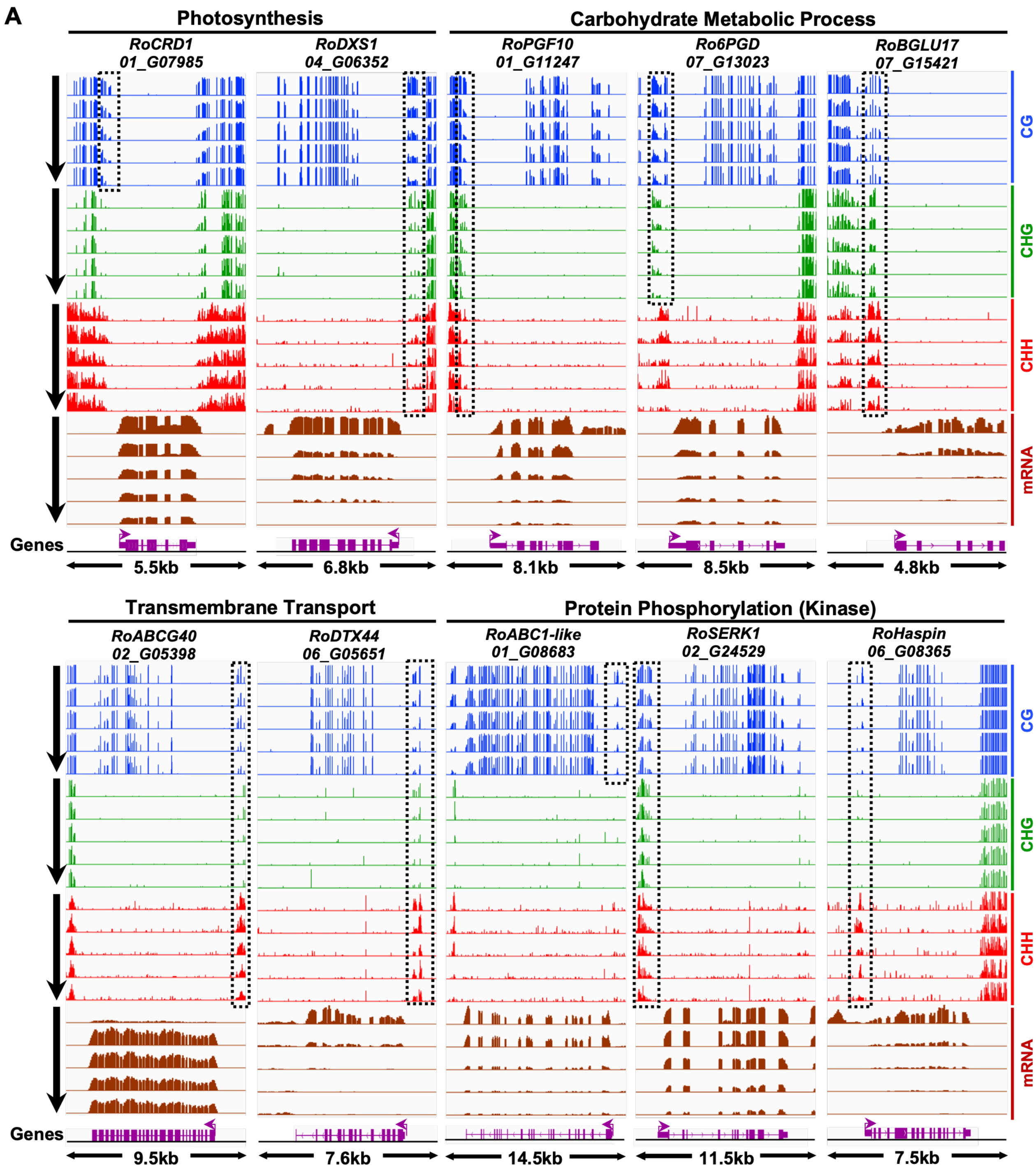

A
Photosynthesis
Carbohydrate Metabolic Process
RoCRD1
01_G07985
RoDXS1
04_G06352
RoPGF10
01_G11247
Ro6PGD
07_G13023
RoBGLU17
07_G15421
CG
CHG
CHH
mRNA
Genes
5.5kb
6.8kb
8.1kb
8.5kb
4.8kb
Transmembrane Transport
Protein Phosphorylation (Kinase)
RoABCG40
02_G05398
RoDTX44
06_G05651
RoABC1-like
01_G08683
RoSERK1
02_G24529
RoHaspin
06_G08365
9.5kb
7.6kb
14.5kb
11.5kb
7.5kb

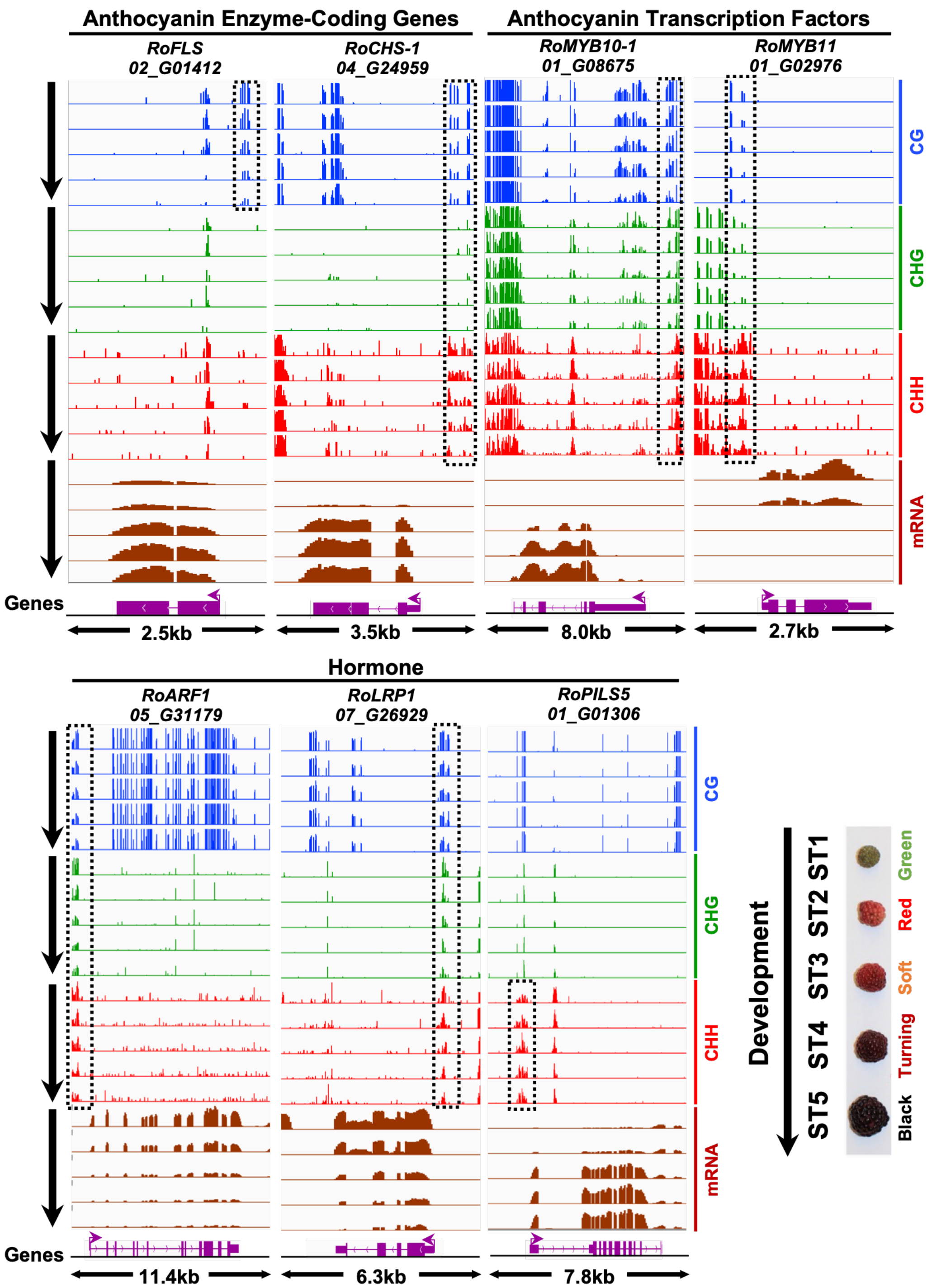
Anthocyanin Enzyme-Coding Genes
Anthocyanin Transcription Factors
RoFLS
02_G01412
RoCHS-1
04_G24959
RoMYB10-1
01_G08675
RoMYB11
01_G02976
CG
CHG
CHH
mRNA
Genes
2.5kb
3.5kb
8.0kb
2.7kb
Hormone
RoARF1
05_G31179
RoLRP1
07_G26929
RoPILS5
01_G01306
11.4kb
6.3kb
7.8kb
Development
ST1
ST2
ST3
ST4
ST5
Green
Red
Soft
Turning
Black

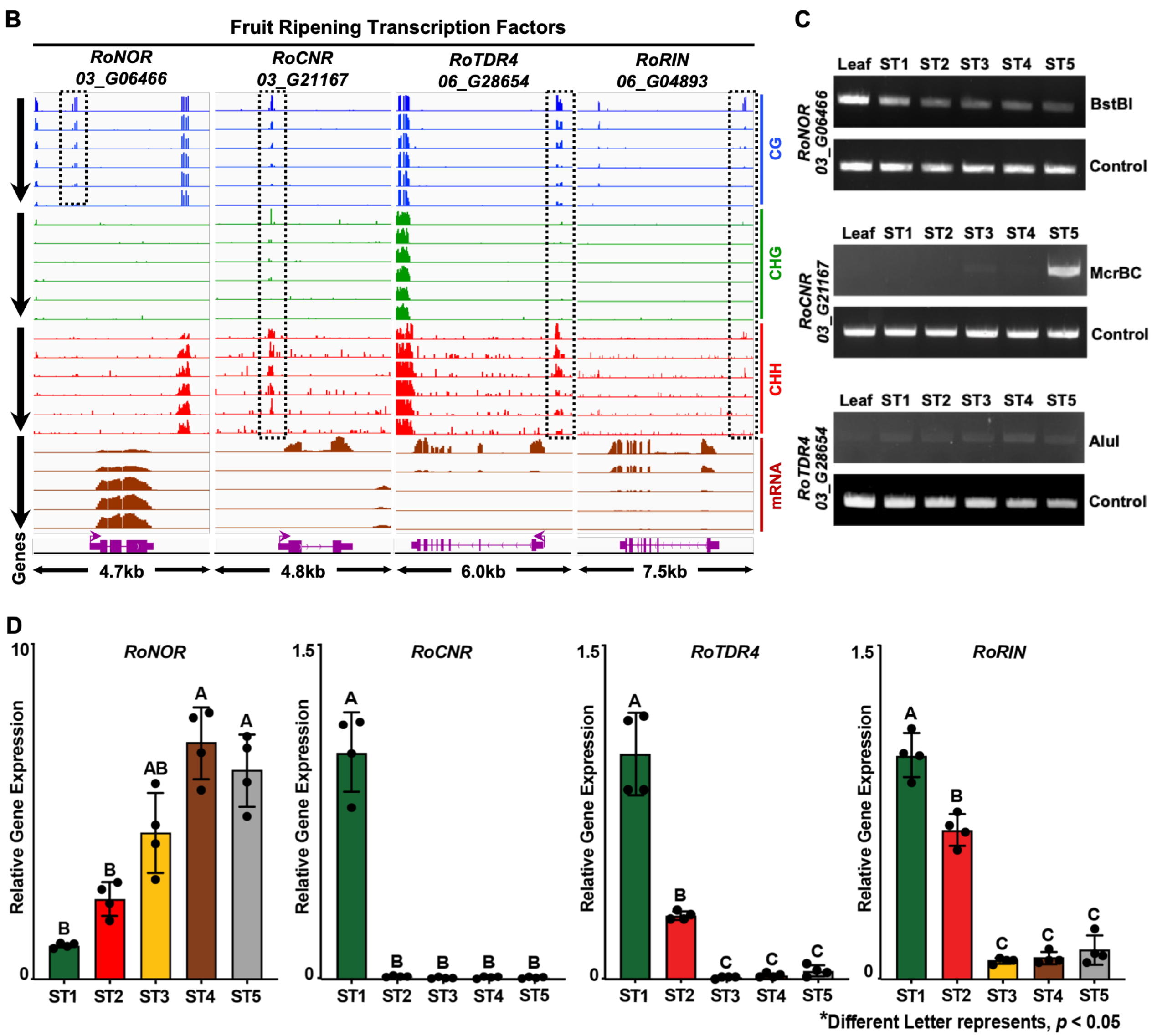

Supplementary Figure 9 | Methylation levels and mRNA accumulation patterns of gene classes during black raspberry fruit ripening. A Integrative Genomics Viewer (IGV) images of enriched genes detected in GO analysis, anthocyanin enzyme-coding genes and anthocyanin-related transcription factors. The arrow indicates the development of fruit ripening stages from ST1 to ST5. **B** IGV images of fruit ripening transcription factors, *RoNOR*, *RoCNR*, *RoTDR4* and *RoRIN*. In addition to fruit stages (ST1 to ST5), DNA methylation at the leaf stage is shown in the first row of each context. **C** Chop-PCR assays reveal the DNA methylation level of *RoNOR*, *RoCNR* and *RoTDR4* across leaf and five fruit ripening stages. BstBI digestion assesses CG sites (blocked by methylation). McrBC digestion cuts all methylated sequences. AluI digestion assesses CHH methylation (hindered but not completely blocked by methylation). **D** The qRT-PCR analysis reveal the relative gene

expression of *RoNOR*, *RoCNR*, *RoTDR4* and *RoRIN* across five fruit ripening stages. *Different letters: one way ANOVA, $p < 0.05$.

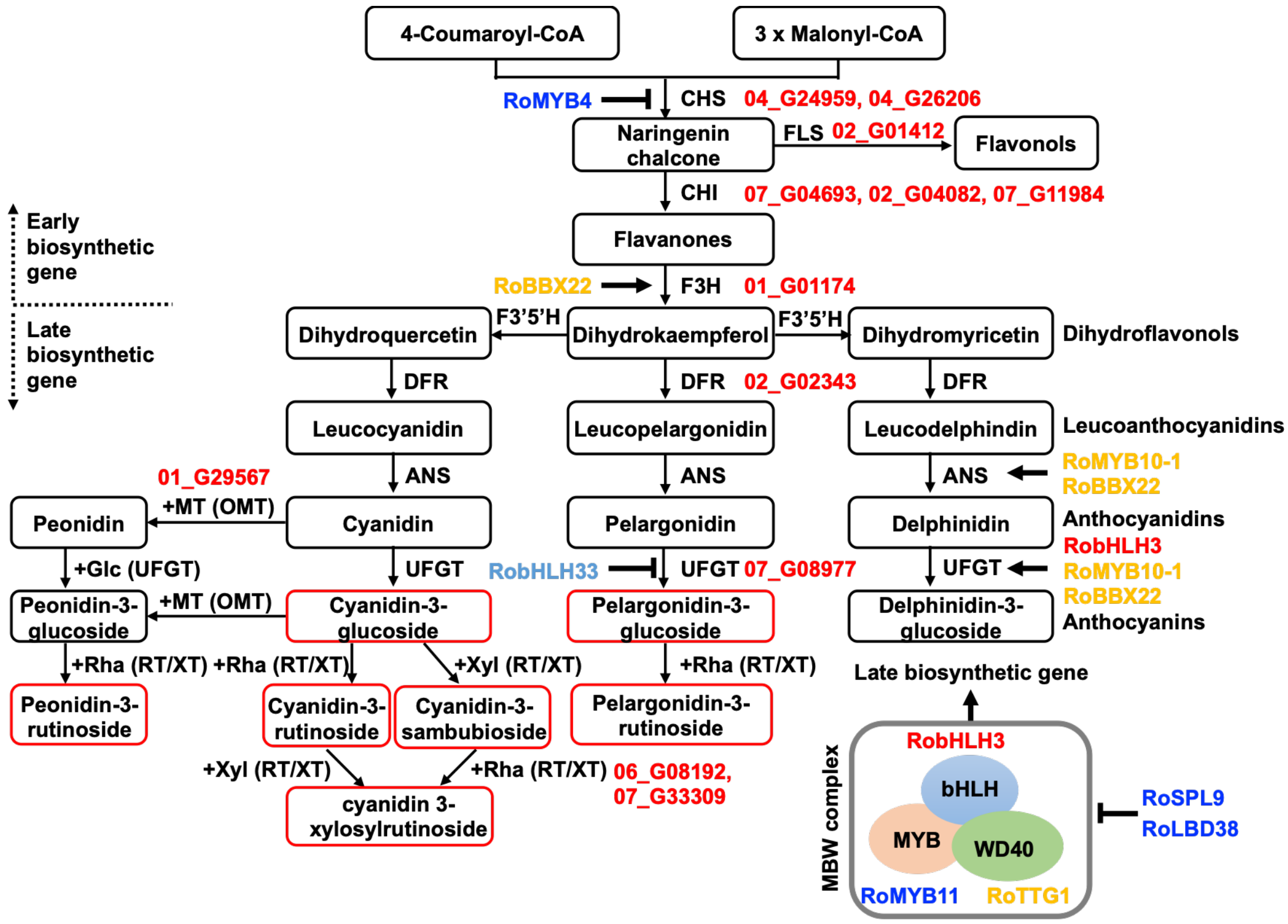

Supplementary Figure 10 | Diagram of the anthocyanin biosynthetic pathway in black raspberry fruit. Gene symbols are color-coded based on the Pearson correlation between transcript abundance and relative anthocyanin content across developmental stages. Red indicates strong positive correlation ($r \geq 0.8$), and blue indicates strong negative correlation ($r \leq -0.8$). Orange and light blue indicate moderate positive ($0.6 \leq r < 0.8$) and moderate negative ($-0.8 < r \leq -0.6$) correlations, respectively.

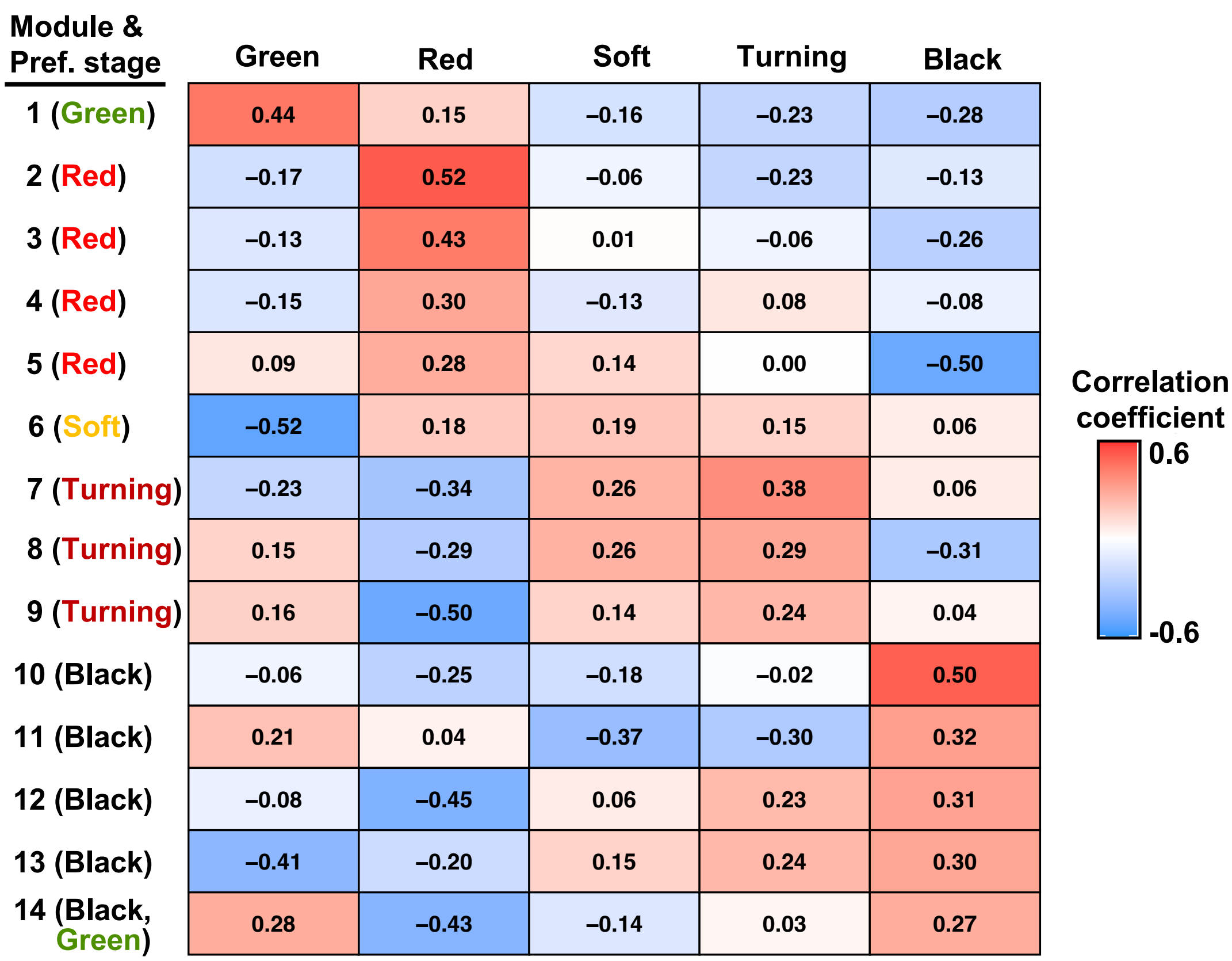

Supplementary Figure 11 | Gene regulatory modules inferred by WGCNA. The heat map illustrates the strength and direction of correlation between module eigengenes and black raspberry fruit ripening stages. For each module, correlation coefficients were calculated by assessing the relationship between transcript abundance patterns and developmental time points, allowing identification of modules associated with specific fruit ripening stages.

A

Chromosome	Heterochromatin	Euchromatin	Chromosome	Heterochromatin	Euchromatin
Chr. 1	17,550,051 - 21,084,501	2,000,001 - 4,750,001	Chr. 5	22,535,801 - 26,022,251	2,040,501 - 4,223,151
Chr. 2	22,203,451 - 25,285,801	5,891,251 - 8,577,201	Chr. 6	46,816,651 - 50,507,801	22,753,151 - 26,311,501
Chr. 3	16,575,051 - 20,744,351	35,637,801 - 39,319,201	Chr. 7	17,415,001 - 22,861,801	36,764,651 - 39,821,851
Chr. 4	18,013,101 - 21,662,751	251,701 - 3,108,901			

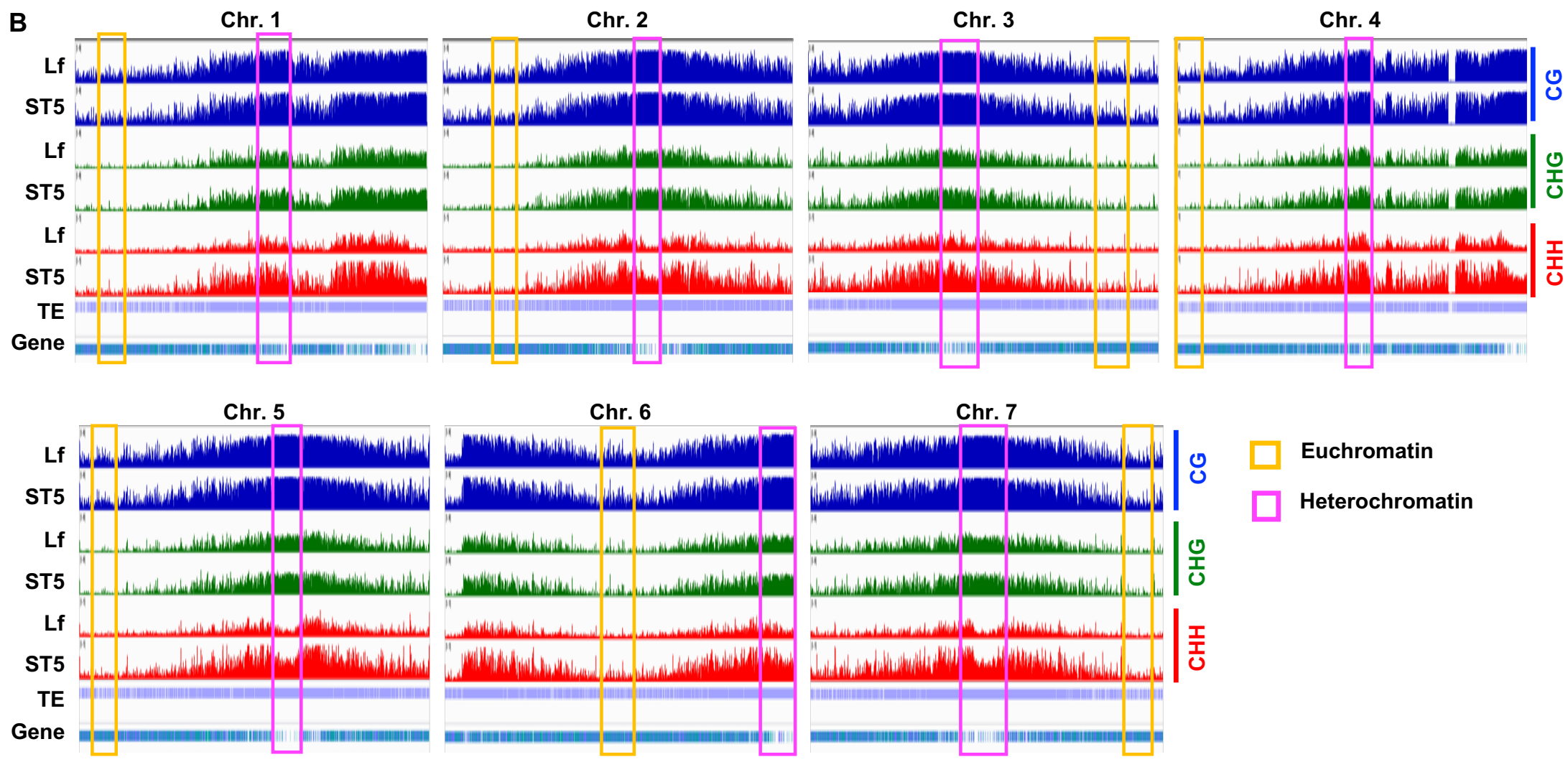

Supplementary Figure 12 | DNA methylation profiling across seven chromosomes of black raspberry in leaf and black-stage fruit tissues. A Approximately 2 to 4 Mb genomic intervals from both heterochromatic (transposon-rich) and euchromatic (gene-rich) regions were randomly selected to calculate DNA methylation levels using 50-bp sliding windows, as shown in Fig. 5. **B** Integrative Genomics Viewer of the randomly selected chromosomal segments. Pink and yellow boxes indicate heterochromatic and euchromatic regions, respectively, which were used for finer-resolution methylation analysis based on 50-bp windows. Lf, leaf; TE, transposable element.
